# Genetic mutations driving ciprofloxacin resistance in laboratory-evolved *Salmonella* Typhimurium

**DOI:** 10.1128/spectrum.01194-26

**Published:** 2026-09-08

**Authors:** Nakjun Choi, Eunna Choi, Yong-Joon Cho, Yajing Wen, Jiangban Zhao, Aidong Han, Eun-Jin Lee

**Affiliations:** 1 Department of Life Sciences, School of Life Sciences and Biotechnology, Korea University 124654, Seoul, South Korea; 2 Department of Molecular Bioscience, Multidimensional Genomics Research Center, College of Biomedical Science, Kangwon National University 235004, Chuncheon-si, South Korea; 3 Department of Biochemistry and Molecular Biology, School of Life Sciences, Xiamen University, Xiang’an 199198, Xiamen, China; Newcastle University, Newcastle Upon Tyne, United Kingdom

**Keywords:** *Salmonella *Typhimurium, ciprofloxacin resistance, efflux pump, TCA cycle, DNA gyrase, whole-genome sequencing

## Abstract

Ciprofloxacin resistance in *Salmonella* Typhimurium is a significant public health concern, and the mechanisms by which the resistance evolves are poorly defined. Here, by serial passaging under antibiotic selection, we isolated ciprofloxacin-resistant *S*. Typhimurium mutants and subjected them to whole-genome sequencing to reveal the major mutations associated with resistance. The Low Cip^R^ mutant acquired four chromosomal mutations in *ramR, icdA, lipB,* and *gyrA*, and the High Cip^R^ mutant gained additional mutations in *gyrB, yaiC,* and *corA*. Functional characterization determined that mutations in *ramR* resulted in efflux pump upregulation, while disruptions in the TCA cycle caused by mutations in *icdA* and *lipB* led to metabolic alterations. These changes indirectly enhanced resistance by increasing the expression of the global regulator MarA and reducing OmpF-dependent membrane permeability. Despite the observation of the G105A substitution in GyrA, enzymatic assays confirmed the failure to support resistance to ciprofloxacin, possibly because the structural alteration remained minimal. GyrB^488–489dup^ was associated with maintained supercoiling under ciprofloxacin and enhanced fluoroquinolone resistance, suggesting a major role in resistance evolution. Other mutations in *yaiC* impaired biofilm and, in *corA*, intracellular accumulation of magnesium, possibly stabilizing the bacterial cell envelope under antibiotic pressure. The findings provide novel explanations for the multifaceted mechanisms leading to ciprofloxacin resistance in *Salmonella* and suggest targets to combat antimicrobial resistance.

**IMPORTANCE:**

Antibiotic resistance in *Salmonella* Typhimurium is an increasing public health concern, yet the genetic changes that allow bacteria to become resistant are not fully understood. In this study, we evolved ciprofloxacin-resistant *Salmonella* in the laboratory and identified the mutations that arise during resistance development. We found that resistance does not result from a single change but from multiple adaptations affecting drug efflux, metabolism, and the antibiotic target. Some mutations increased the activity of pumps that remove antibiotics from the cell, while others altered bacterial metabolism and reduced membrane permeability, making it harder for the drug to enter. A duplication in the DNA gyrase subunit GyrB played a particularly important role in maintaining DNA function under antibiotic stress. Together, these results reveal how diverse genetic changes cooperate to generate ciprofloxacin resistance and provide insights that may help guide strategies to combat drug-resistant *Salmonella* infections.

## INTRODUCTION

*Salmonella* Typhimurium is a gram-negative bacterium belonging to the Enterobacteriaceae family and is a major foodborne pathogen responsible for gastroenteritis in humans and animals. Transmission primarily occurs through the consumption of contaminated food or cross-contamination (1, 2). While *Salmonella* infections are often self-limiting in immunocompetent individuals, antibiotic treatment is essential for immunocompromised patients, infants, the elderly, and those with severe infections (3). Fluoroquinolones, cephalosporins, and azithromycin are commonly used therapeutic options (4). However, the increasing prevalence of antibiotic-resistant *Salmonella* strains has made treatment more challenging, raising serious public health concerns (5, 6). In addition, the widespread use of antibiotics in livestock and poultry farming has been recognized as a major driver of antimicrobial resistance, as *S*. Typhimurium remains a critical zoonotic threat, with multidrug-resistant strains increasingly reported in farm animals and poultry (7–9). From a One Health perspective, the emergence and spread of resistant *Salmonella* at the animal–human–environment interface emphasize the need to understand the molecular mechanisms underlying resistance.

Ciprofloxacin, a fluoroquinolone antibiotic, exhibits strong activity against both gram-positive and gram-negative bacteria and is frequently used to treat bacterial infections caused by *Salmonella* species, *Escherichia coli*, and *Pseudomonas aeruginosa* (10). It inhibits DNA gyrase and topoisomerase IV, blocking bacterial DNA replication and leading to cell death (11). Due to its efficacy, ciprofloxacin has been a first-line treatment for severe *Salmonella* infections (12). However, recent epidemiological studies report a global increase in ciprofloxacin-resistant *Salmonella* strains, particularly in hospitals and foodborne isolates. This resistance is associated with increased treatment failures, prolonged hospitalizations, and the persistence of resistant strains in food production systems (13, 14).

Ciprofloxacin resistance primarily arises through mutations in genes encoding DNA gyrase and topoisomerase IV (e.g., *gyrA* and *gyrB*), upregulation of efflux pump components (*acrAB-tolC*), and acquisition of plasmid-mediated quinolone resistance (the *qnr* family) (11, 15, 16). To investigate the molecular mechanisms underlying ciprofloxacin resistance, we generated laboratory-evolved ciprofloxacin-resistant *Salmonella* strains through experimental evolution via serial passaging under gradually increasing ciprofloxacin concentrations as a selective pressure, thereby driving the accumulation of adaptive mutations. We then identified key genetic mutations through whole-genome sequencing. The ciprofloxacin-resistant *Salmonella* strains harbored mutations in *ramR*, *lipB*, *icdA*, and *gyrA* in the Low Cip^R^ mutant, while additional mutations in *yaiC*, *gyrB*, and *corA* were observed in the High Cip^R^ mutant. These mutations span regulatory, metabolic, and antibiotic target genes, suggesting multiple pathways contribute to ciprofloxacin resistance.

## RESULTS

### Generating mutations in *Salmonella* strain to confer resistance to ciprofloxacin

**Fig 1:**
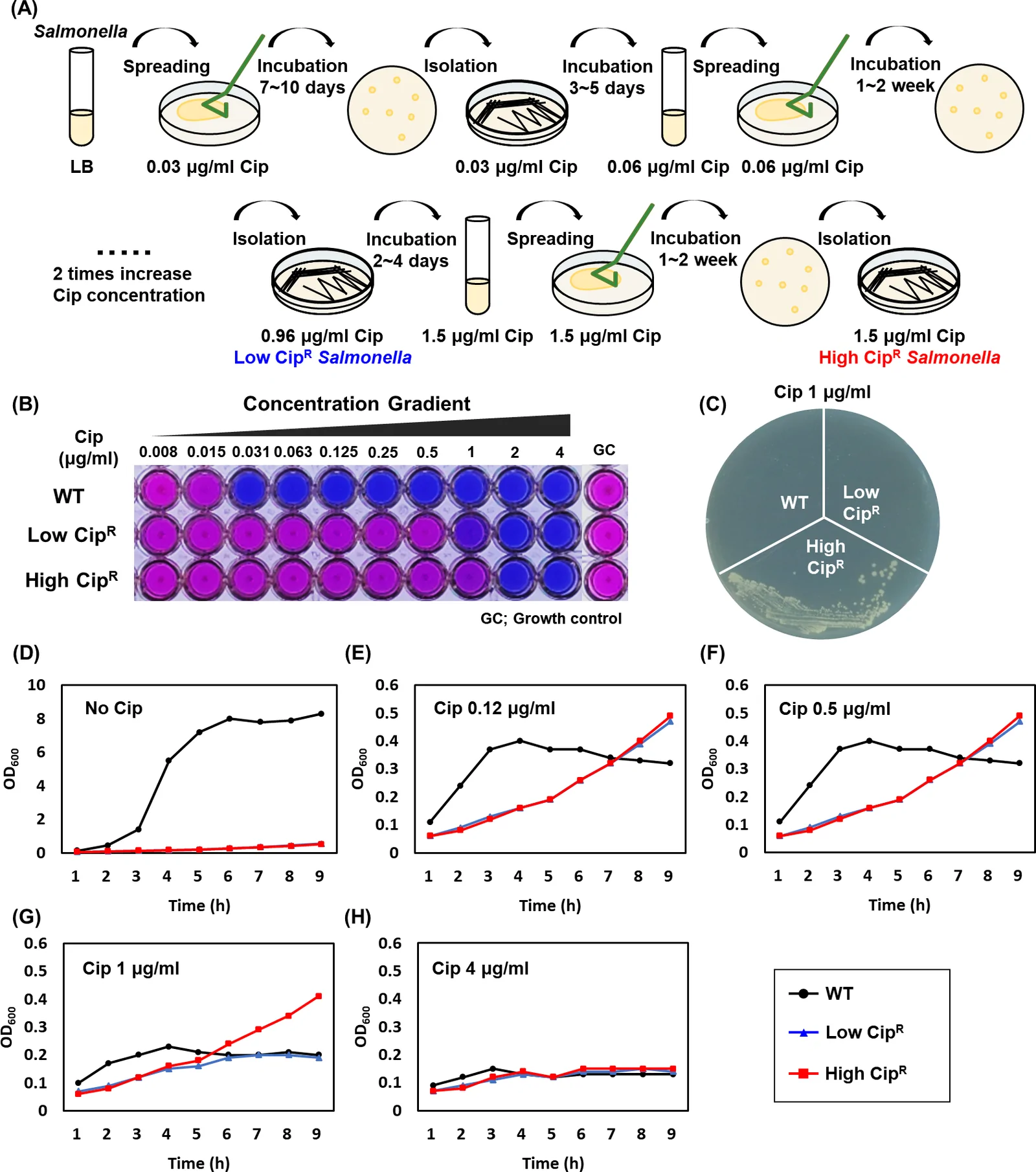
Ciprofloxacin-resistant *Salmonella* strains were generated in the laboratory. (**A**) Schematic diagram illustrating the generation of ciprofloxacin-resistant *Salmonella* strains. (**B**) Minimum inhibitory concentration assay for ciprofloxacin performed using 96-well plates with wild-type 14028s, Low Cip^R^, and High Cip^R^ strains. The strains were cultured in Mueller-Hinton broth. After incubation, 0.015% resazurin dye was added. Pink indicates bacterial growth, while blue indicates no bacterial growth. (**C**) Wild-type, Low Cip^R^, and High Cip^R^ *Salmonella* strains streaked on an LB plate containing 1 μg/mL ciprofloxacin. (**D–H**) Growth curves of wild-type, Low Cip^R^, and High Cip^R^ *Salmonella* strains grown in LB liquid media with different concentrations of ciprofloxacin, as indicated.

To generate ciprofloxacin-resistant *Salmonella enterica* serovar Typhimurium 14028s strains, the wild-type strain was serially passaged in gradually increasing concentrations of ciprofloxacin, starting at the wild-type MIC (0.03 μg/mL) (Fig. 1A). A surviving colony isolated on Luria-Bertani (LB) agar containing 0.96 μg/mL ciprofloxacin was designated Low Cip^R^, and subsequent selection at 1.5 μg/mL ciprofloxacin yielded the High Cip^R^ strain.

To evaluate ciprofloxacin resistance of the Cip^R^ mutants, we performed a minimum inhibitory concentration (MIC) assay and confirmed stepwise increases in ciprofloxacin resistance (Fig. 1B). The MICs for the Low Cip^R^ and High Cip^R^ strains were 1 and 2 μg/mL, respectively, corresponding to 32- and 64-fold increases relative to the wild type (0.031 μg/mL). On LB agar containing 1 μg/mL ciprofloxacin, the wild-type strain failed to grow, whereas the Low Cip^R^ strain formed small colonies and the High Cip^R^ strain exhibited normal colony morphology (Fig. 1C). According to CLSI M100 guidelines (17), both Cip^R^ strains were classified as ciprofloxacin resistant.

Growth analyses in LB medium containing increasing ciprofloxacin concentrations further supported resistance phenotypes (Fig. 1D). The Cip^R^ mutants showed greater growth than the wild type at 0.12 and 0.5 μg/mL ciprofloxacin, whereas only the High Cip^R^ mutant exhibited substantial growth at 1 μg/mL ciprofloxacin. No strain grew at 4 μg/mL ciprofloxacin.

### Identification of mutations leading to ciprofloxacin resistance in the Low and High Cip^R^ strains

**Fig 2:**
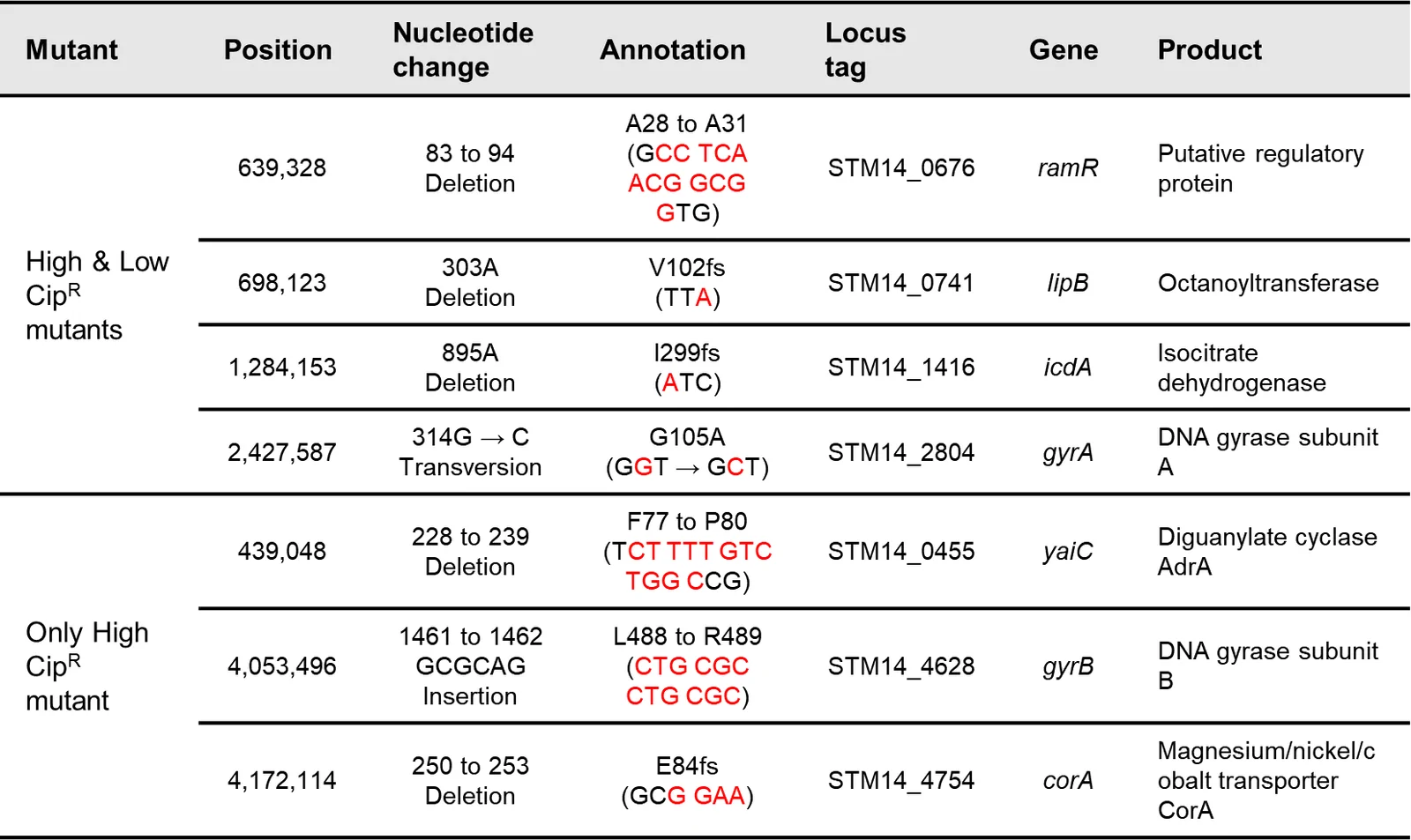
Location of mutations in the Low and High ciprofloxacin-resistant *Salmonella* strains. These mutations were identified by whole-genome sequencing and filtered for nucleotide changes that resulted in amino acid substitutions. Specifically, mutations with a reference position value >1,000 and a forward/reverse balance >0.4 were considered.

To identify the mutations responsible for conferring ciprofloxacin resistance to the wild type, we performed whole-genome sequencing on the Cip^R^ mutants (Fig. 2). The Low Cip^R^ mutant harbored four mutations in *ramR*, *lipB*, *icdA*, and *gyrA*, while the High Cip^R^ mutant carried three additional mutations in *yaiC*, *gyrB*, and *corA*.

In the Low Cip^R^ mutant, the *ramR* gene, which encodes the regulatory protein RamR, contained a 12-nucleotide deletion (CCTCAACGGCGG) spanning positions 83–94, resulting in a truncated form, RamR^Δ28–31^. The *lipB* gene, encoding octanoyl transferase, exhibited a frameshift mutation caused by an adenine deletion at nucleotide position 303, producing LipB^V102fs^. Similarly, the *icdA* gene, encoding isocitrate dehydrogenase, had a frameshift mutation due to an adenine deletion at nucleotide position 895, generating IcdA^I299fs^. The *gyrA* gene, encoding DNA gyrase subunit A, displayed a G-to-C transversion at nucleotide position 314 (GGT→GCT), substituting glycine at position 105 with alanine, thereby generating GyrA^G105A^.

The High Cip^R^ mutant, in addition to the mutations identified in the Low Cip^R^ strain, harbored three unique mutations. The *yaiC* gene, encoding the diguanylate cyclase AdrA, had a 12-nucleotide deletion (CTTTTGTCTGGC) spanning positions 228–239, resulting in YaiC^Δ77–80^. The *gyrB* gene, encoding DNA gyrase subunit B, exhibited a six-nucleotide insertion (CTGCGC) at nucleotide positions 1462–1467, producing GyrB^488–489dup^. Finally, the *corA* gene, encoding the magnesium transporter CorA, contained a four-nucleotide deletion (GGAA) at nucleotide positions 250–253, leading to a frameshift mutation that resulted in a truncated form, CorA^E84fs^.

### The *ramR* mutation in ciprofloxacin-resistant *Salmonella* leads to constitutive expression of the efflux pump

**Fig 3:**
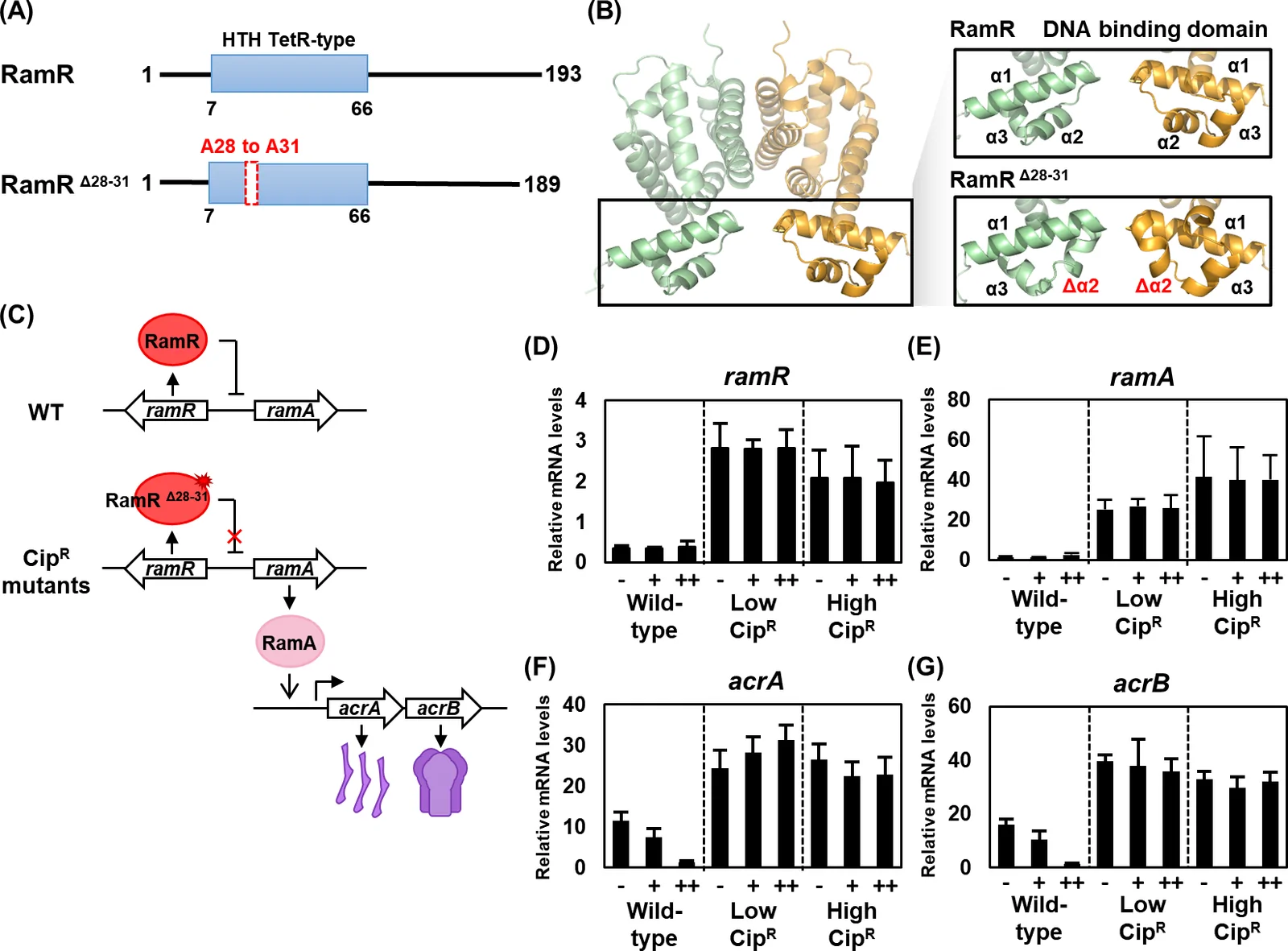
The *ramR* mutation in ciprofloxacin-resistant *Salmonella* strains upregulates the transcription of multidrug efflux genes. (**A**) The *ramR* mutation results in the deletion of amino acids 28–31 in the RamR transcription regulator (RamR^Δ28–31^). (**B**) Modeled structures of RamR and RamR^Δ28–31^, highlighting the DNA-binding domain region. The deleted region in RamR^Δ28–31^ is part of the DNA-binding domain, and this deletion is predicted to weaken DNA binding. The modeled structure of the mutated proteins was generated using AlphaFold 3 (ipTM = 0.7 and pTM = 0.71). (**C**) Diagram illustrating the potential regulatory mechanism of *ramR*^Δ28–31^ in Cip^R^ mutants. (**D–G**) Relative mRNA levels of *ramR, ramA, acrA*, and *acrB* genes in wild-type, Low Cip^R^, and High Cip^R^ strains. Cells were grown in LB in the presence or absence of ciprofloxacin (+, 0.12 μg/mL; ++, 0.5 μg/mL). The mRNA levels of *ramR, ramA, acrA,* and *acrB* were determined by real-time quantitative reverse transcription PCR. Mean values and standard deviations were calculated from three independent experiments.

The transcriptional regulator RamR is a helix-turn-helix (HTH)-type repressor that inhibits the expression of *ramA*, a transcriptional activator that promotes the expression of AcrAB-TolC efflux pump components AcrA and AcrB (18). In the Cip^R^ mutants, a deletion of four amino acids (A28–A31) within the HTH domain was identified (Fig. 3A), disrupting the α2 helix, a critical component of the DNA-binding domain (Fig. 3B). As a result, RamR^Δ28–31^ was expected to fail to suppress *ramA* expression, leading to constitutive production of the AcrAB efflux pump subunits AcrA and AcrB under the regulation of RamA (Fig. 3C).

To test this hypothesis, we performed real-time quantitative reverse transcription PCR (qRT-PCR) to measure the expression levels of the *ramR*, *ramA*, *acrA*, and *acrB* genes (Fig. 3D through G). In the wild-type strain, the expression levels of *ramR* and *ramA* remained unchanged regardless of ciprofloxacin treatment. In contrast, their expression elevated significantly in the Cip^R^ mutants independent of ciprofloxacin exposure. Additionally, the expression of *acrA* and *acrB* decreased in a dose-dependent manner in the wild-type strain, whereas the Cip^R^ mutants displayed consistently high expression of both genes regardless of ciprofloxacin treatment. Thus, the Cip^R^ mutants exhibited constitutive overexpression of the AcrAB-TolC efflux pump, regardless of the presence of ciprofloxacin, providing a robust mechanism of antibiotic resistance.

### Mutations in *lipB* and *icdA* disrupt the TCA cycle, leading to metabolic stress and redox imbalance that enhance ciprofloxacin resistance via efflux pump activation

**Fig 4:**
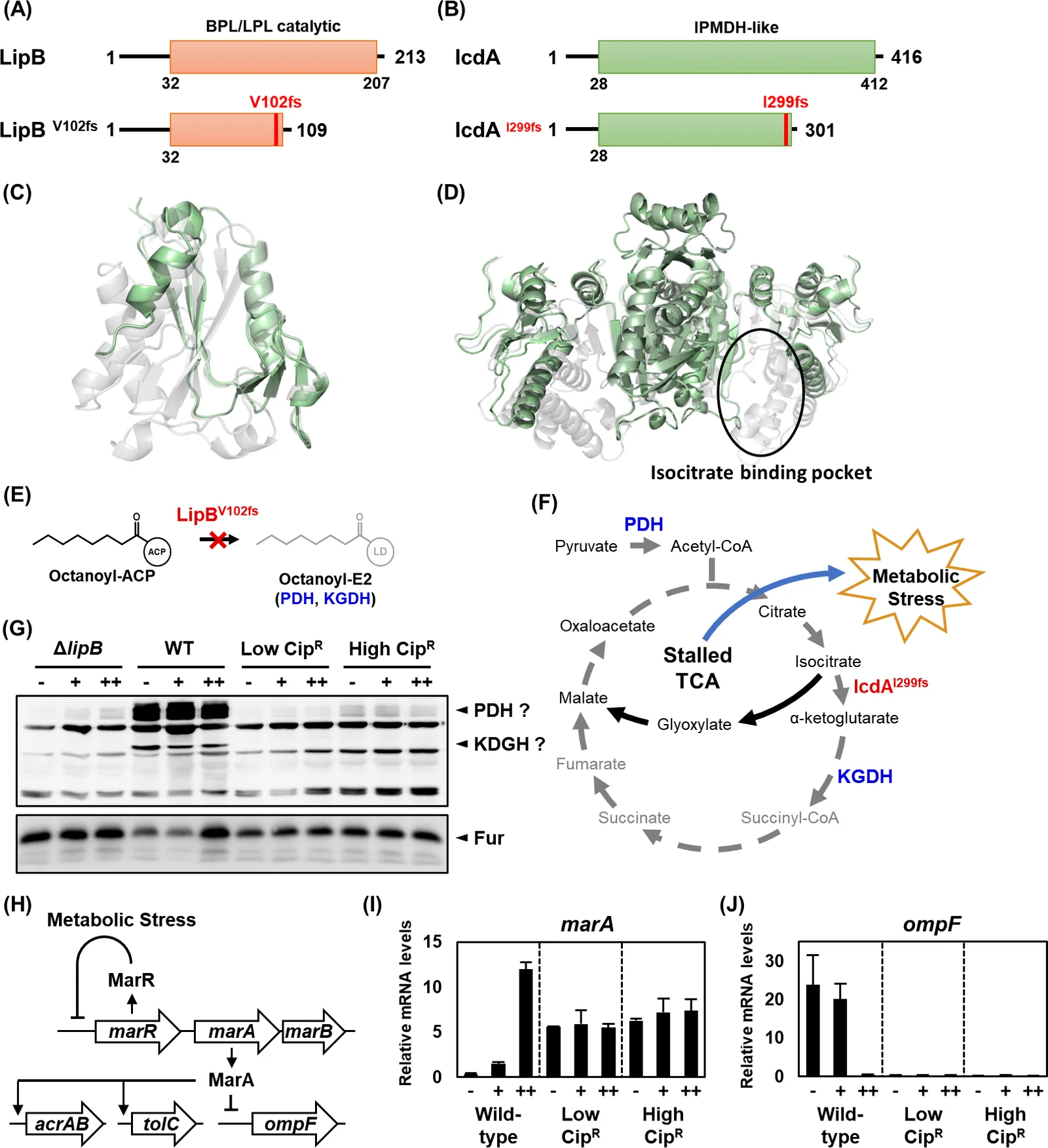
Mutations in *lipB* and *icdA* lead to a stalled TCA cycle. (**A and B**) *lipB* and *icdA* mutations result in a frameshift at the 102^nd^ amino acid in LipB and the 299^th^ amino acid in IcdA, respectively. (**C and D**) Modeled structures of LipB and IcdA proteins. The original structures of LipB and IcdA are colored in light gray. The *icdA* mutation removes the isocitrate-binding pocket, possibly affecting the metabolic pathway. The modeled protein structures were generated using AlphaFold 3 (pTM = 0.84 for LipB^V102fs^, ipTM = 0.81, and pTM = 0.82 for IcdA^I299fs^). (**E**) The *lipB* frameshift mutation in Cip^R^ mutants may affect the original function of LipB, which transfers the octanoyl group to the E2 subunits of PDH and KGDH. (**F**) Diagram illustrating a potential TCA cycle in Cip^R^ mutants. Nonfunctional mutations in *lipB* and *icdA* disrupt the functions of key enzymes in the TCA cycle, likely causing the cycle to stall. (**G**) Western blot analysis of crude extracts from wild-type, Low Cip^R^, High Cip^R^, and Δ*lipB* mutant strains. Blots were probed with anti-lipoic acid and anti-Fur antibodies to detect the octanoyl-E2 subunits of PDH and KGDH, and Fur proteins, respectively. Bacteria were grown for 4.5 h in LB or LB supplemented with ciprofloxacin (+, 0.12 μg/mL; ++, 0.5 μg/mL), as described in Materials and Methods. (**H**) Schematic diagram of *marA* expression by metabolic stress and subsequent *ompF* repression. (**I and J**) Relative mRNA levels of *marA* and *ompF* genes in wild-type, Low Cip^R^, and High Cip^R^ strains. Bacterial cells were grown in LB with ciprofloxacin (+, 0.12 μg/mL; ++, 0.5 μg/mL). The *marA* and *ompF* mRNA levels were determined by qRT-PCR. Mean values and standard deviations from three independent experiments are shown.

LipB is an octanoyl transferase that delivers octanoyl residues essential for the function of the pyruvate dehydrogenase (PDH) and α-ketoglutarate dehydrogenase (KGDH) complexes, which are critical components of the TCA cycle (19, 20). IcdA, on the other hand, is an isocitrate dehydrogenase that catalyzes the oxidative decarboxylation of isocitrate to α-ketoglutarate within the TCA cycle (21). In the Cip^R^ mutants, the LipB^V102fs^ protein is truncated due to a frameshift mutation after valine at position 102, resulting in a prematurely terminated 109-amino-acid protein that lacks the full catalytic domain (Fig. 4A). Because LipB^V102fs^ is approximately half the length of the wild-type LipB, it is presumed to be nonfunctional (Fig. 4C and E). Consistent with this, western blot analysis using an anti-lipoic acid antibody revealed the absence of the octanoyl-E2 band in the Δ*lipB* mutant as well as in the Low Cip^R^ and High Cip^R^ mutants (Fig. 4G). Similarly, the IcdA^L299fs^ protein was truncated after isoleucine at position 299, producing a 301-amino-acid protein that lacks a functional isocitrate-binding pocket (Fig. 4B and D). The loss of these critical structural elements renders LipB^V102fs^ and IcdA^I299fs^ nonfunctional, impairing their respective enzymatic roles in the TCA cycle (Fig. 4F).

Disruption of the TCA cycle can induce metabolic stress and activate MarA, a regulator of multidrug resistance that promotes the AcrAB-TolC efflux pump while repressing the porin OmpF (Fig. 4H) (22–24). To investigate these effects, we measured *marA* and *ompF* expression by qRT-PCR. In the wild-type strain, *marA* expression increased and *ompF* expression decreased in response to ciprofloxacin concentrations. In contrast, the Cip^R^ mutants displayed constitutively high *marA* expression and markedly reduced *ompF* expression regardless of ciprofloxacin exposure (Fig. 4I and J). These findings suggest that *lipB* and *icdA* mutations impair the TCA cycle, inducing metabolic stress that enhances efflux and reduces membrane permeability, thereby reinforcing ciprofloxacin resistance.

### DNA gyrase mutations reduce DNA supercoiling efficiency but increase ciprofloxacin resistance

**Fig 5:**
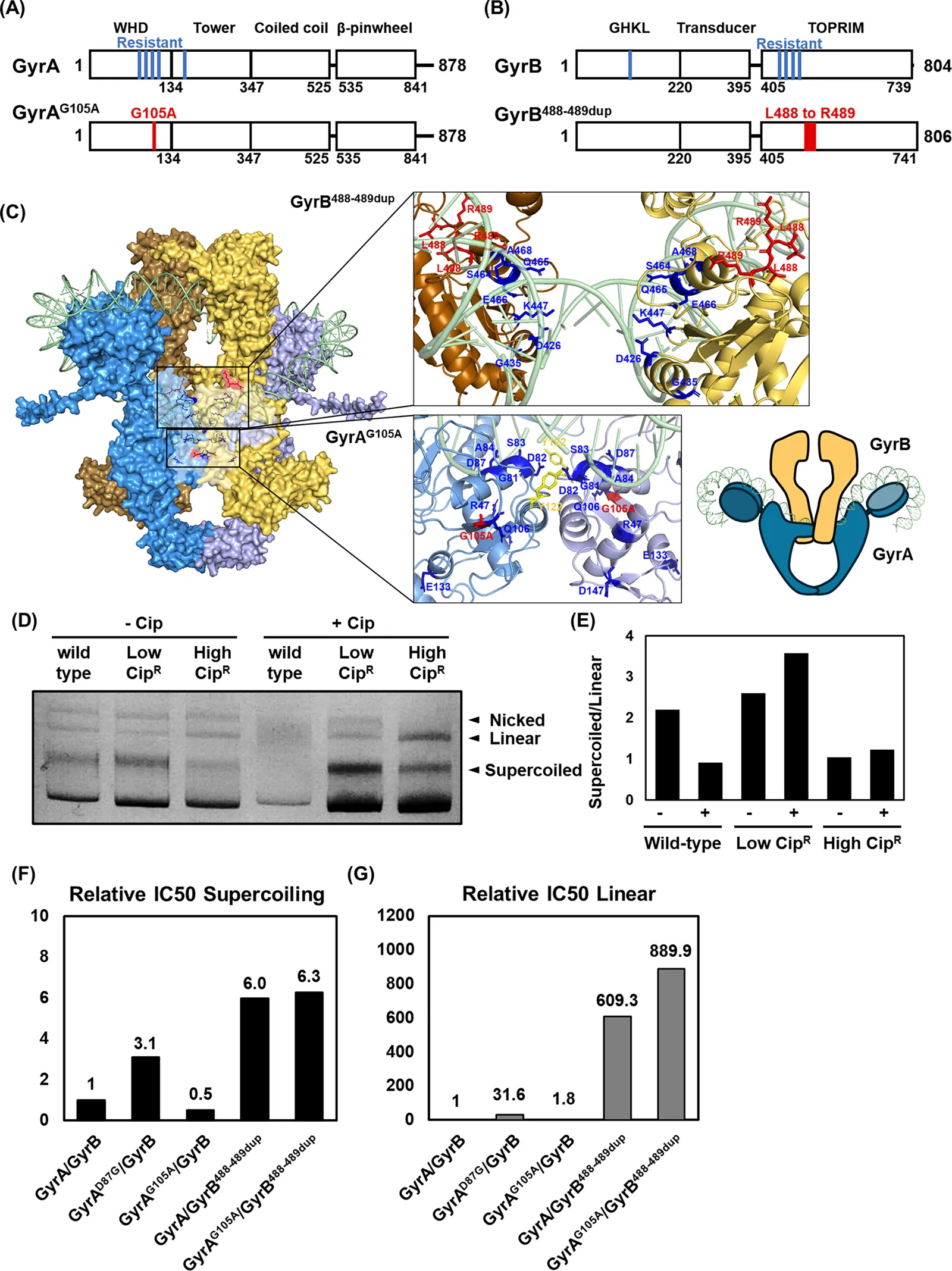
Mutations in *gyrA* and *gyrB* decrease ciprofloxacin susceptibility by increasing DNA supercoiling despite the presence of ciprofloxacin. (**A and B**) Domain schemes of GyrA and GyrB. The *gyrA* mutation results in an amino acid substitution at position 105, replacing glycine with alanine (G105A), while the *gyrB* mutation results in a duplication of leucine and arginine at positions 488 and 489 (488–489dup). Blue vertical lines indicate the locations of residues previously identified as required for ciprofloxacin resistance. (**C**) Modeled structures of GyrA and GyrB. Residues highlighted in blue represent amino acids previously identified as critical for fluoroquinolone resistance, whereas residues in red represent mutations identified in this study. The DNA structure is shown in light green. The modeled protein complex structures were generated using AlphaFold 3 (ipTM = 0.64 and pTM = 0.66 for the GyrA–GyrB complex). (**D and E**) Effects of ciprofloxacin on DNA supercoiling. The pBR322 plasmid was introduced into wild-type and Cip^R^ mutant strains. Plasmids of 100–200 ng were loaded onto a 1.5% agarose gel for electrophoresis. The intensity of DNA bands was quantified using ImageJ software, and the degree of DNA supercoiling was calculated as the supercoiled-to-linear ratio. (**F and G**) Cleavage complex assay with moxifloxacin. Gyrase heterotetramers (A_2_B_2_, 50 nM) containing wild type or the mutations GyrA^D87G^, GyrA^G105A^, and GyrB^488–489dup^ were assembled from purified subunits. (**F**) Supercoiled fraction IC_50_. (**G**) Linear fraction IC_50_. IC_50_ values are shown as fold change relative to wild-type gyrase. Dose-dependent responses were fitted by nonlinear regression and calculated based on the reduction of supercoiled DNA.

Ciprofloxacin directly binds to the GyrA subunit of DNA gyrase, inhibiting its activity and exerting antibacterial effects (25). As a result, ciprofloxacin-resistant strains frequently harbor mutations in DNA gyrase (26). In the Cip^R^ mutants we generated, GyrA carried a substitution of glycine at position 105 with alanine (Fig. 5A and C). In contrast, the High Cip^R^ mutant possessed a duplication of leucine 488 and arginine 489 in the topoisomerase-primase (TOPRIM) domain of GyrB, a region known to play a key role in DNA supercoiling and relaxation activities (Fig. 5B and C).

To determine how these mutations affect DNA supercoiling, we transformed the wild-type and Cip^R^ mutants with the pBR322 plasmid, re-extracted the plasmid, and analyzed it by electrophoresis on a 1.5% agarose gel (Fig. 5D). The band intensities were quantified using ImageJ to calculate the supercoiled-to-linear (SL) DNA ratio (Fig. 5E). In the absence of ciprofloxacin, plasmids extracted from the wild type and Low Cip^R^ mutant predominantly displayed supercoiled and circular forms, with no significant differences. However, plasmids from the High Cip^R^ mutant exhibited a reduced supercoiled band and a more intense linear band (low SL ratio), indicating reduced supercoiling efficiency. Upon ciprofloxacin treatment, plasmids extracted from the wild-type strain lost their supercoiled band and instead showed smeared linear and nicked DNA. In contrast, ciprofloxacin did not alter the DNA supercoiling patterns of the Low and High Cip^R^ mutants.

Because the Low Cip^R^ mutant did not exhibit altered supercoiling patterns despite carrying the GyrA^G105A^ substitution, we hypothesized that this mutation alone does not significantly affect DNA gyrase activity. To test this hypothesis, we performed enzymatic assays to compare the inhibition of plasmid supercoiling by moxifloxacin using reconstituted gyrase variants. The enzymatic inhibition assays showed that GyrA^D87G^, used as a control, exhibited a higher IC_50_ in the supercoiled fraction, whereas Gyr^G105A^ exhibited a lower IC_50_ than the wild type. Similarly, in the linear fraction, GyrA^G105A^ fell between wild type and Gyr^D87G^ but remained close to the wild type. In contrast, GyrB^488–489dup^ displayed a markedly higher IC_50_ value, and the GyrA^G105A^/GyrB^488–489dup^ combination behaved similarly to GyrB^488–489dup^ in both supercoiled and linear fractions (Fig. 5F and G). These findings indicate that although GyrA^G105A^ contributes marginally to ciprofloxacin resistance, GyrB^488–489dup^ significantly enhances fluoroquinolone resistance, likely through reduced supercoiling efficiency. This suggests that the additional GyrB^488–489dup^ mutation in the High Cip^R^ mutant is a key factor contributing to its higher ciprofloxacin resistance compared to the Low Cip^R^ mutant.

### Potential role of *yaiC* and *corA* mutations in ciprofloxacin resistance in the High Cip^R^ mutant

**Fig 6:**
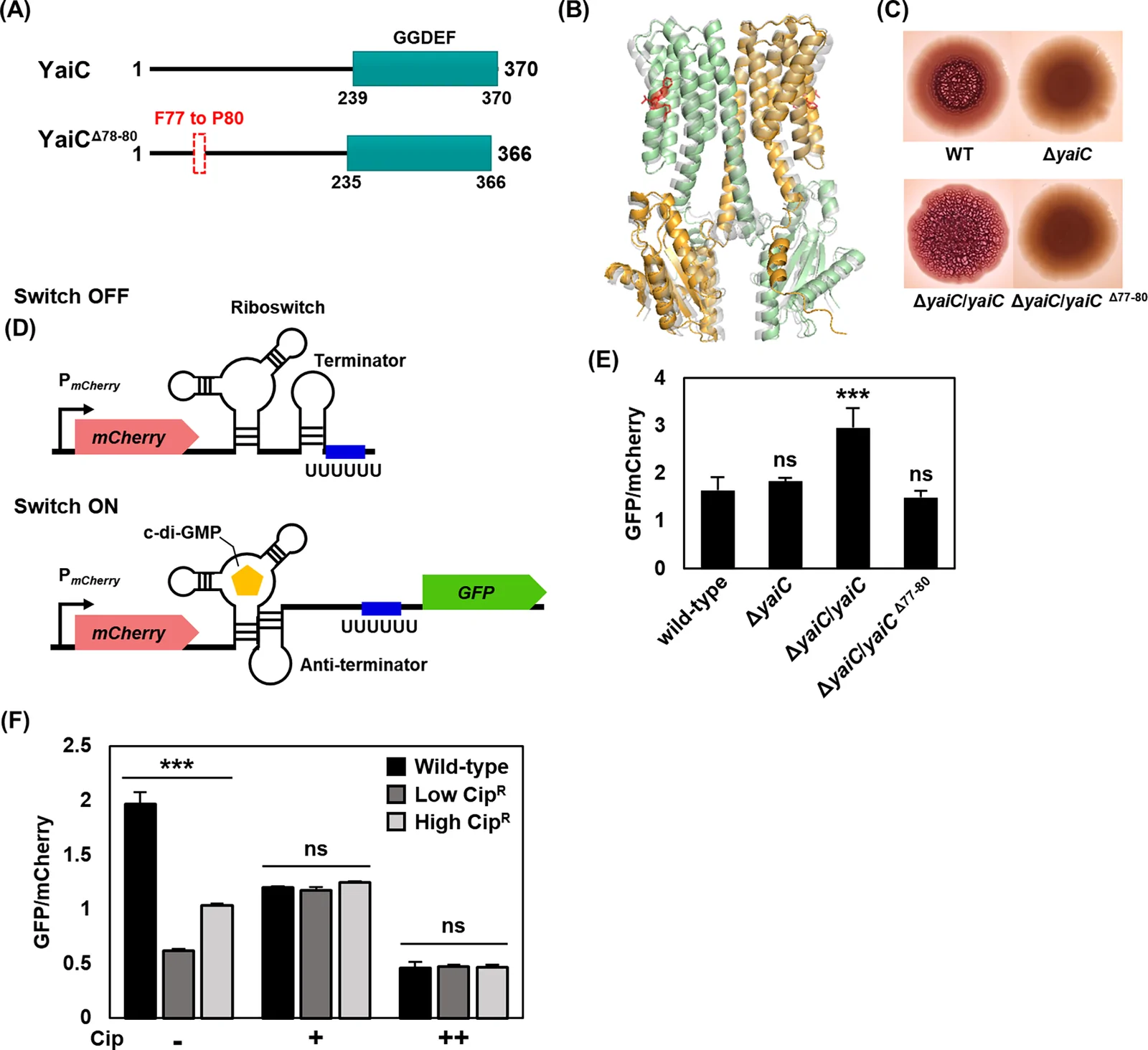
The *yaiC* mutation in the High Cip^R^ mutant impairs biofilm formation and c-di-GMP synthesis. (**A**) The *yaiC* mutation with a deletion of amino acids from 77 to 80. (**B**) Modeled structures of YaiC dimer. The wild-type YaiC is shown in light gray, while the YaiC^Δ77–80^ dimer is superimposed in pale green and bright yellow. The mutation in YaiC shortens the transmembrane alpha helix, with the deleted amino acids highlighted in red. The modeled protein structures were generated using AlphaFold 3 (ipTM = 0.54 and pTM = 0.56). (**C**) The red, dry, and rough morphology assay of Δ*yaiC* mutants and complemented strains. Wild-type/pBAD33, Δ*yaiC*/pBAD33, Δ*yaiC*/pBAD33-*yaiC*, and Δ*yaiC*/pBAD33-*yaiC*^Δ77–80^ strains were spotted on LB agar without NaCl, containing Congo red and Coomassie blue, and incubated at room temperature for 6 days. (**D**) Diagram of c-di-GMP riboswitch. In the presence of c-di-GMP (yellow pentagon), the riboswitch forms a pseudoknot structure that sequesters the transcription terminator, allowing GFP expression. (**E**) Intracellular c-di-GMP levels of the above strains measured using a c-di-GMP riboswitch. yaiC* indicates pBAD33-*yaiC*^Δ77–80^. GFP fluorescence intensity, controlled by the c-di-GMP riboswitch, was measured and normalized to the OD_600_ values. Data represent the means ± SD from three independent experiments. Statistical analysis was performed using one-way ANOVA followed by Dunnett’s multiple comparisons test (***, *P* < 0.001). (**F**) Intracellular c-di-GMP levels of the wild-type, Low Cip^R^, and High Cip^R^ strains with or without ciprofloxacin (+, 0.12 μg/mL; ++, 0.5 μg/mL) treatment. Data represent the means ± SD from three independent experiments. Statistical analysis was performed using two-way ANOVA followed by Tukey’s multiple comparisons test (***, *P* < 0.001).

Only the High Cip^R^ mutant harbored mutations in the *yaiC* and *corA* genes. *yaiC* encodes the diguanylate cyclase AdrA, which is known to play a critical role in biofilm formation by synthesizing the secondary messenger c-di-GMP (27, 28). The High Cip^R^ mutant possesses a 12-nucleotide deletion in *yaiC*, resulting in the truncated protein YaiC^Δ77–80^ (Fig. 6A). This mutation is located in a region that forms part of the membrane helix (Fig. 6B).

To evaluate biofilm formation ability, we performed a red, dry, and rough (RDAR) assay using Congo red agar (29). Biofilm formation was not observed in the High Cip^R^ mutant; interestingly, it was also absent in the Low Cip^R^ mutant, which carries no mutation in *yaiC* (Fig. S1). To assess the functionality of YaiC^Δ77–80^, we generated a Δ*yaiC* mutant, and the wild-type *yaiC* and *yaiC*^Δ77–80^ alleles from the High Cip^R^ mutant were cloned into expression vectors for complementation experiments. RDAR morphology was clearly observed in the wild-type strain and Δ*yaiC* complemented with wild-type *yaiC*. In contrast, neither the Δ*yaiC* mutant nor the Δ*yaiC* mutant complemented with *yaiC*^Δ77–80^ exhibited RDAR morphology (Fig. 6C).

To determine whether the YaiC^Δ77–80^ mutation affects biofilm formation through impaired interaction with BscB, a cellulose synthase subunit (27), or through loss of c-di-GMP production, we performed an assay using a riboswitch-based c-di-GMP sensor (Fig. 6D and E). While c-di-GMP levels were similar between the wild-type and Δ*yaiC* strains, c-di-GMP levels significantly increased when the Δ*yaiC* mutant was complemented with wild-type *yaiC*. However, complementation with *yaiC*^Δ77–80^ from the High Cip^R^ mutant did not restore c-di-GMP levels, indicating that the c-di-GMP synthesis activity of YaiC^Δ77–80^ was impaired.

We next examined the effects of ciprofloxacin on c-di-GMP levels. In the wild-type strain, c-di-GMP levels decreased as ciprofloxacin concentration increased. Both Cip^R^ mutants exhibited lower basal c-di-GMP levels than the wild-type strain in the absence of ciprofloxacin, and upon drug exposure, reporter levels did not decline further and were comparable to the wild type (Fig. 6F).

**Fig 7:**
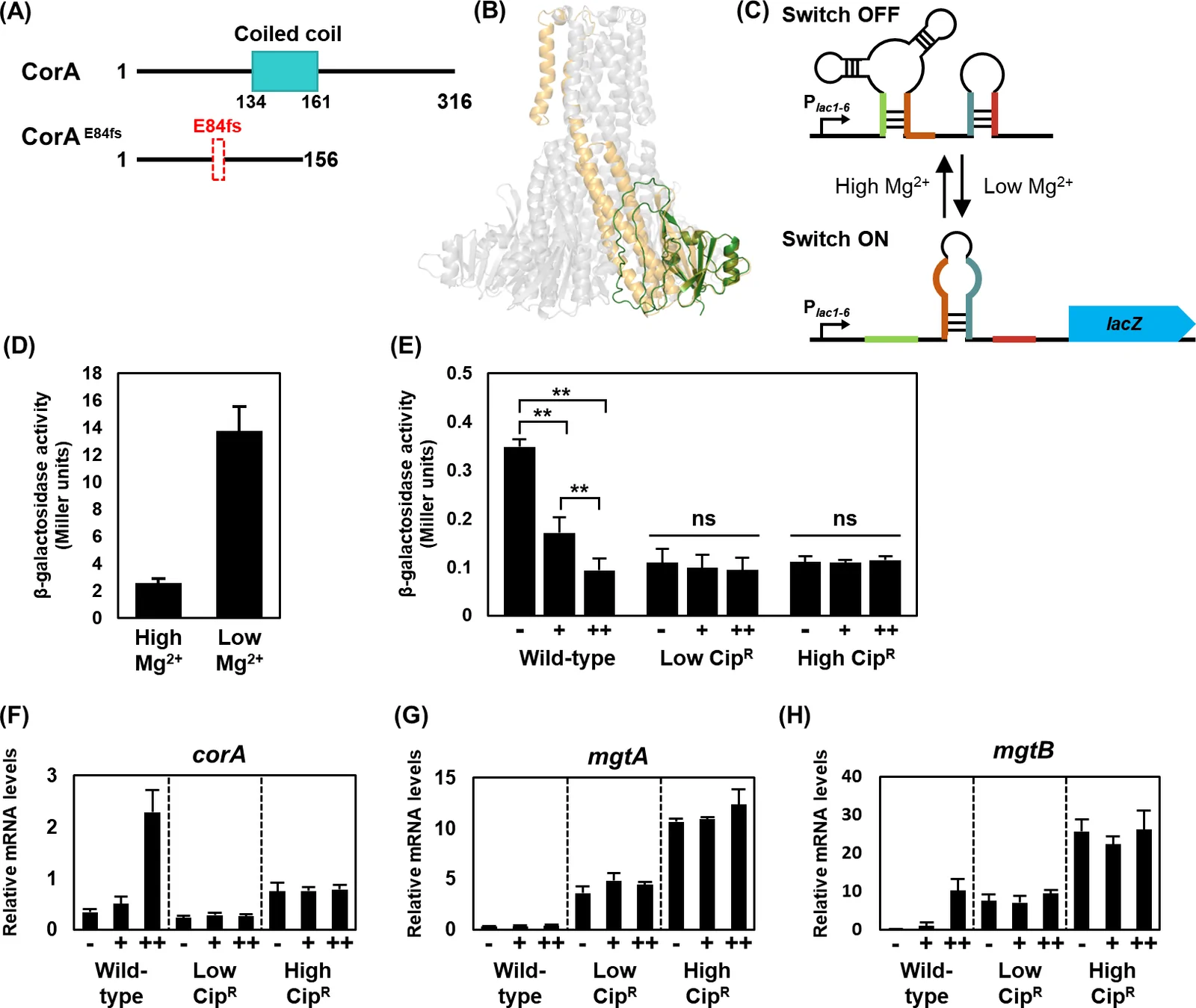
The *corA* mutation in the High Cip^R^ mutant disrupts Mg^2+^ transporter formation and alters intracellular Mg^2+^ levels. (**A**) The *corA* mutation with a frameshift at position 84, resulting in a truncated 156-amino acid protein. (**B**) The modeled structure of the CorA protein pentamer. The wild-type CorA pentamer is shown in light gray, with the CorA monomer depicted in transparent bright orange. The truncated CorA^E84fs^ monomer is highlighted in pale green. The modeled protein structures were generated using AlphaFold 3 (pTM = 0.52). (**C**) Diagram of Mg^2+^ riboswitch. In low Mg^2+^, the riboswitch forms a secondary structure that sequesters the transcription terminator, allowing *lacZ* expression. (**D**) β-galactosidase activity (Miller units) was measured in the wild-type strain harboring the pYS1010 plasmid, which contains the Mg^2+^-responsive *mgtA* 5′ UTR fused to the *lacZ* gene under the p*_lac1-6_* promoter. Strains were grown in N-minimal medium containing either 10 mM or 10 µM Mg^2+^ for 24 h. (**E**) β-galactosidase activity was measured in wild-type, Low Cip^R^, and High Cip^R^ strains carrying the pYS1010 plasmid grown for 4.5 h in LB with ciprofloxacin (+, 0.12 μg/mL; ++, 0.5 μg/mL). Data represent the means ± SD from three independent experiments. Statistical analysis was performed using two-way ANOVA followed by Tukey’s multiple comparisons test (**, *P* < 0.002). (**F–H**) Relative mRNA levels of *corA*, *mgtA*, and *mgtB* genes in wild-type, Low Cip^R^, and High Cip^R^ strains grown in LB medium with ciprofloxacin (+, 0.12 μg/mL; ++, 0.5 μg/mL) were measured by qRT-PCR. Mean values and standard deviations were calculated from three independent experiments.

Another unique mutation identified in the High Cip^R^ mutant was in the *corA* gene, which encodes the magnesium transporter CorA. The High Cip^R^ mutant harbored a four-nucleotide deletion in *corA*, leading to a frameshift mutation after glutamate at position 84. As a result, the CorA protein lost its original sequence beyond glutamate 84, producing a truncated protein of 156 amino acids (Fig. 7A). This truncation lacks domains required for pentamer assembly and Mg^2+^ channel formation (Fig. 7B).

To assess the effect of CorA^E84fs^ and the resulting changes in intracellular Mg^2+^ concentration, we transformed the wild-type, Δ*corA*, Low Cip^R^, and High Cip^R^ strains with the pYS1010 plasmid, which contains the *mgtA* 5′ UTR riboswitch fused to the *lacZ* reporter (Fig. 7C) (30). These strains were grown in N-minimal medium containing either high (10 mM) or low (10 μM) Mg^2+^ concentrations, and β-galactosidase activity was measured using *o*-nitrophenyl-β-D-galactopyranoside (ONPG). In the wild-type strain, β-galactosidase activity increased significantly in N-minimal medium containing 10 μM Mg^2+^ compared to 10 mM Mg^2+^ (Fig. 7D), indicating that the Mg^2+^ riboswitch reflects intracellular Mg^2+^ levels. We next assessed whether ciprofloxacin modulates intracellular Mg^2+^. In the wild type, increasing ciprofloxacin concentrations led to a progressive decrease in reporter output, indicating a relative increase in intracellular Mg^2+^ under drug exposure. In contrast, both Cip^R^ mutants showed low reporter activity at baseline and little additional change with ciprofloxacin treatment, consistent with a Mg^2+^-replete state irrespective of drug exposure (Fig. 7E).

To further investigate magnesium transporter expressions, we measured the mRNA levels of *corA*, *mgtA*, and *mgtB* using qRT-PCR (Fig. 7F through H). In the wild-type strain, *corA* mRNA levels increased with ciprofloxacin treatment, whereas the Low Cip^R^ mutant exhibited consistently low *corA* expression regardless of ciprofloxacin exposure. The High Cip^R^ mutant showed minimal changes in *corA* expression (Fig. 7F). For *mgtA*, the wild type displayed consistently low mRNA levels independent of ciprofloxacin treatment. In contrast, the Low Cip^R^ mutant maintained moderate *mgtA* expression, while the High Cip^R^ mutant exhibited consistently high *mgtA* expression regardless of ciprofloxacin concentration (Fig. 7G). *mgtB* mRNA levels showed a notable increase in the wild-type strain at 0.5 µg/mL ciprofloxacin. In the Low Cip^R^ mutant, *mgtB* expression remained high irrespective of ciprofloxacin exposure. Notably, the High Cip^R^ mutant displayed more than twofold higher *mgtB* mRNA levels than the Low Cip^R^ mutant, independent of ciprofloxacin treatment (Fig. 7H).

### The Cip^R^ mutants show reduced invasion and replication inside macrophages but are unaffected by ciprofloxacin treatment

**Fig 8:**
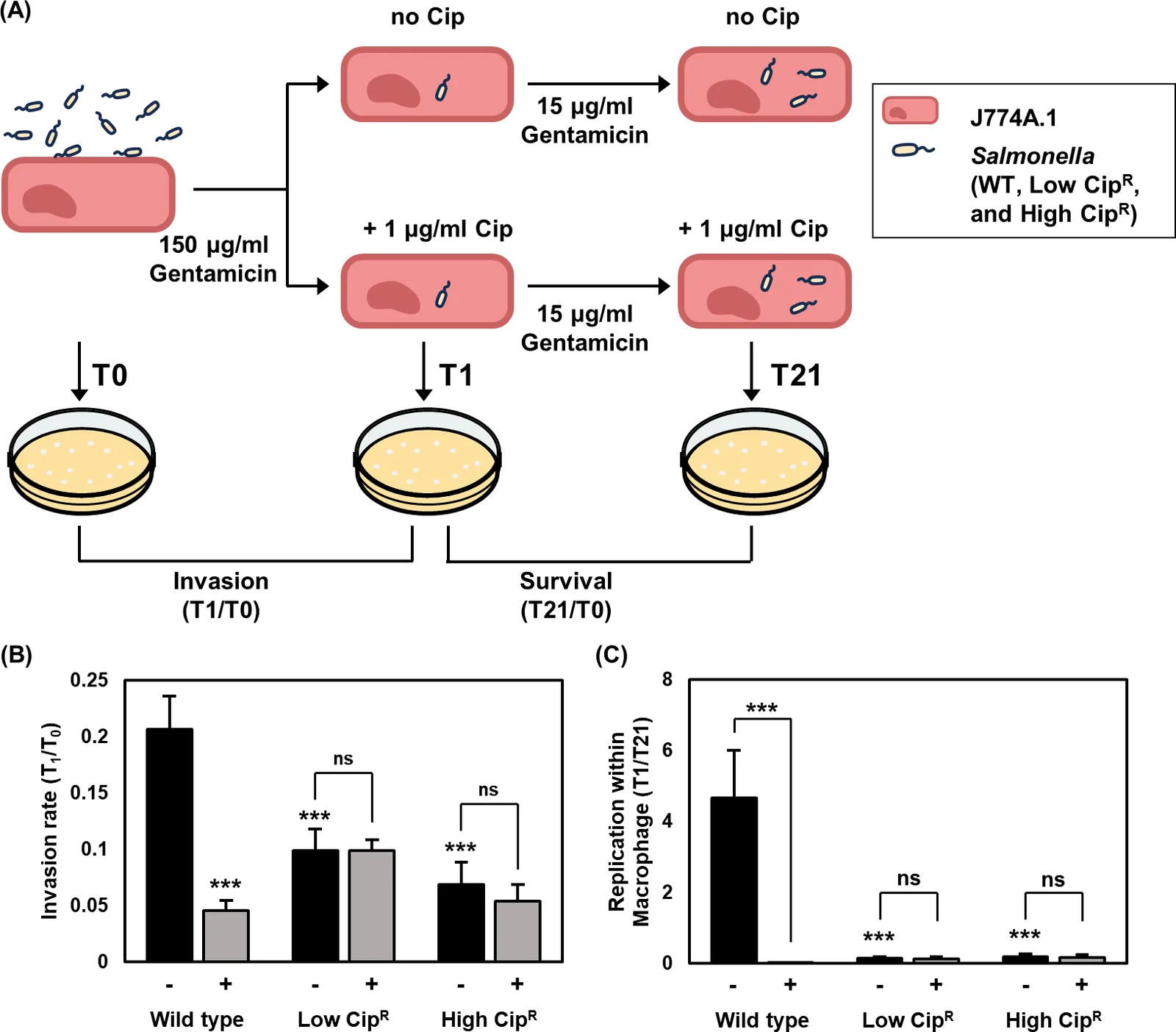
The Cip^R^ mutants do not further decrease macrophage survival after ciprofloxacin treatment. (**A**) Schematic representation of gentamicin protection assay of wild-type, Low Cip^R^, and High Cip^R^ *Salmonella* strains using the J774A.1 macrophage cell line. (**B**) Invasion efficiency into macrophages was assessed in the presence or absence of ciprofloxacin (−, 0 μg/mL; +, 1 μg/mL). The Cip^R^ mutants exhibited significantly lower invasion efficiency compared to the wild type (*P* < 0.001). Upon ciprofloxacin treatment, the invasion efficiency of the wild type decreased significantly (*P* < 0.001), whereas the Cip^R^ mutants showed no further reduction. (**C**) Replication efficiency within macrophages was also assessed. The Cip^R^ mutants exhibited significantly reduced replication efficiency compared to the wild type (wild type, 4.6; Low Cip^R^, 0.14; and High Cip^R^, 0.18; *P* < 0.001). Upon ciprofloxacin treatment, the replication efficiency of the wild type decreased dramatically (0.0079; *P* < 0.001). In contrast, the replication efficiency of the Cip^R^ mutants was not significantly affected. Data represent the means ± SD from three independent experiments. Statistical analysis was performed using two-way ANOVA followed by Tukey’s multiple comparisons test (***, *P* < 0.001).

To evaluate the virulence of Cip^R^ mutants and their ability to invade and replicate within macrophages, a gentamicin protection assay was performed using the J774A.1 macrophage-like cell line (Fig. 8A) (31). Invasion efficiency was first assessed in the presence and absence of ciprofloxacin (1 μg/mL). The Cip^R^ mutants exhibited significantly lower invasion rates than the wild type under normal conditions. However, upon ciprofloxacin treatment, the invasion rate of the wild type decreased dramatically, whereas the Cip^R^ mutants showed no significant reduction, indicating their resistance to the inhibitory effects of ciprofloxacin on invasion (Fig. 8B).

Next, replication rates within macrophages were analyzed. The Cip^R^ mutants demonstrated significantly reduced replication efficiency compared to the wild-type strain. In response to ciprofloxacin treatment, replication of the wild-type strain was completely abolished. In contrast, the replication rates of the Cip^R^ mutants remained unchanged, further highlighting their insensitivity to ciprofloxacin (Fig. 8C). These results suggest that Cip^R^ mutants exhibit reduced virulence, as evidenced by lower invasion and replication rates within macrophages. However, their ability to resist ciprofloxacin allows them to maintain intracellular survival under antibiotic pressure.

### Low Cip^R^ shows potential for multidrug resistance, while High Cip^R^ exhibits collateral sensitivity

To investigate whether Cip^R^ mutants induced by ciprofloxacin developed multidrug resistance, we measured the MICs of ampicillin (Amp), kanamycin (Kan), chloramphenicol (Chl), tetracycline (Tet), trimethoprim (Tmp), and nitrofurantoin (Nif) (Table 1). While ciprofloxacin resistance was markedly increased in the Cip^R^ mutants, resistance to the other antibiotics showed only moderate changes.

**TABLE 1:** MICs of Cip^R^ *Salmonella* strains for various antibiotics^*a*^

Strains	MIC (μg/mL)						
	Cip	Amp	Kan	Chl	Tet	Tmp	Nif
Wild type	0.06	2	8	16	2	2	32
Low Cip^R^	1	4	16	32	16	16	16
High Cip^R^	2	4	16	16	4	2	8

^*a*^ MIC assay to confirm the resistance of wild type and Cip^R^ mutants to different antibiotics for multidrug resistance. Cip, ciprofloxacin; Amp, ampicillin; Kan, kanamycin; Chl, chloramphenicol; Tet, tetracycline; Tmp, trimethoprim; and Nif, nitrofurantoin.

The Low Cip^R^ mutant exhibited modest increases in resistance to Amp, Kan, and Chl (twofold relative to the wild type) but showed larger increases in resistance to Tet and Tmp (eightfold) (Table 1). In contrast, susceptibility to Nif increased, with the MIC reduced by half relative to the wild-type strain. The High Cip^R^ mutant exhibited similar MICs for Amp and Kan, whereas resistance to Chl, Tet, and Tmp was reduced compared to the Low Cip^R^ mutant, approaching wild-type levels. Notably, Nif susceptibility further increased in the High Cip^R^ mutant, with a fourfold lower MIC than the wild-type strain.

These findings suggest that while the Low Cip^R^ mutant shows potential for multidrug resistance, the additional mutations in the High Cip^R^ mutant contribute to collateral sensitivity, rendering it more susceptible to certain antibiotics despite its higher levels of ciprofloxacin resistance.

### The loss-of-function genes in Cip^R^ mutants contribute differently to ciprofloxacin resistance

**Fig 9:**
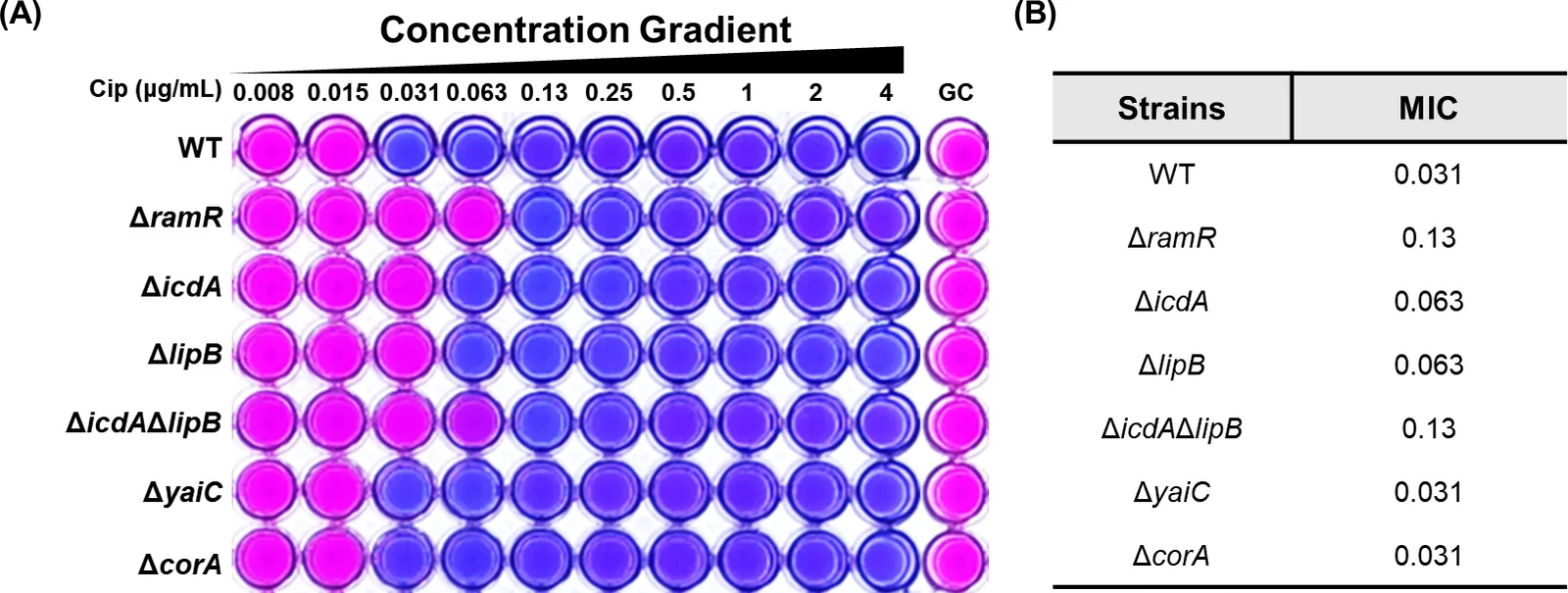
Deletion of *ramR*, *icdA*, or *lipB* contributes to ciprofloxacin resistance. (**A and B**) Minimal inhibitory concentration assays for ciprofloxacin were performed using 96-well plates with the wild-type*,* Δ*ramR,* Δ*icdA,* Δ*lipB,* Δ*icdA*Δ*lipB,* Δ*yaiC,* and Δ*corA* strains. After incubation, 0.015% resazurin dye was added. MIC assays were performed in duplicate for each strain.

To assess the contribution of relevant genes in Cip^R^ mutants to ciprofloxacin resistance, we generated *ramR*, *icdA*, *lipB*, *yaiC*, and *corA* knockout mutants. In addition, to evaluate the combined effects of TCA cycle disruption and magnesium transport on ciprofloxacin resistance, we constructed an *icdA* and *lipB* double knockout mutant and performed a ciprofloxacin MIC assay (Fig. 9).

The MIC for ciprofloxacin in the Δ*ramR* mutant was 0.13 μg/mL, representing a fourfold increase compared to the wild type. In the Δ*icdA* and Δ*lipB* mutants, the MIC increased twofold to 0.063 μg/mL, whereas the Δ*icdA*Δ*lipB* double knockout mutant exhibited a fourfold increase to 0.13 μg/mL. In contrast, the MIC values for the Δ*yaiC* and Δ*corA* knockout mutants remained identical to the wild type (0.031 μg/mL).

Thus, the *ramR* mutation significantly increased ciprofloxacin resistance, with additional contributions from TCA cycle disruption. In contrast, among the additional mutations present in the High Cip^R^ mutant, only the *gyrB* mutation directly contributed to increasing the ciprofloxacin MIC, whereas the *yaiC* and *corA* mutations likely mitigated the physiological costs associated with high-level resistance.

## DISCUSSION

**Fig 10:**
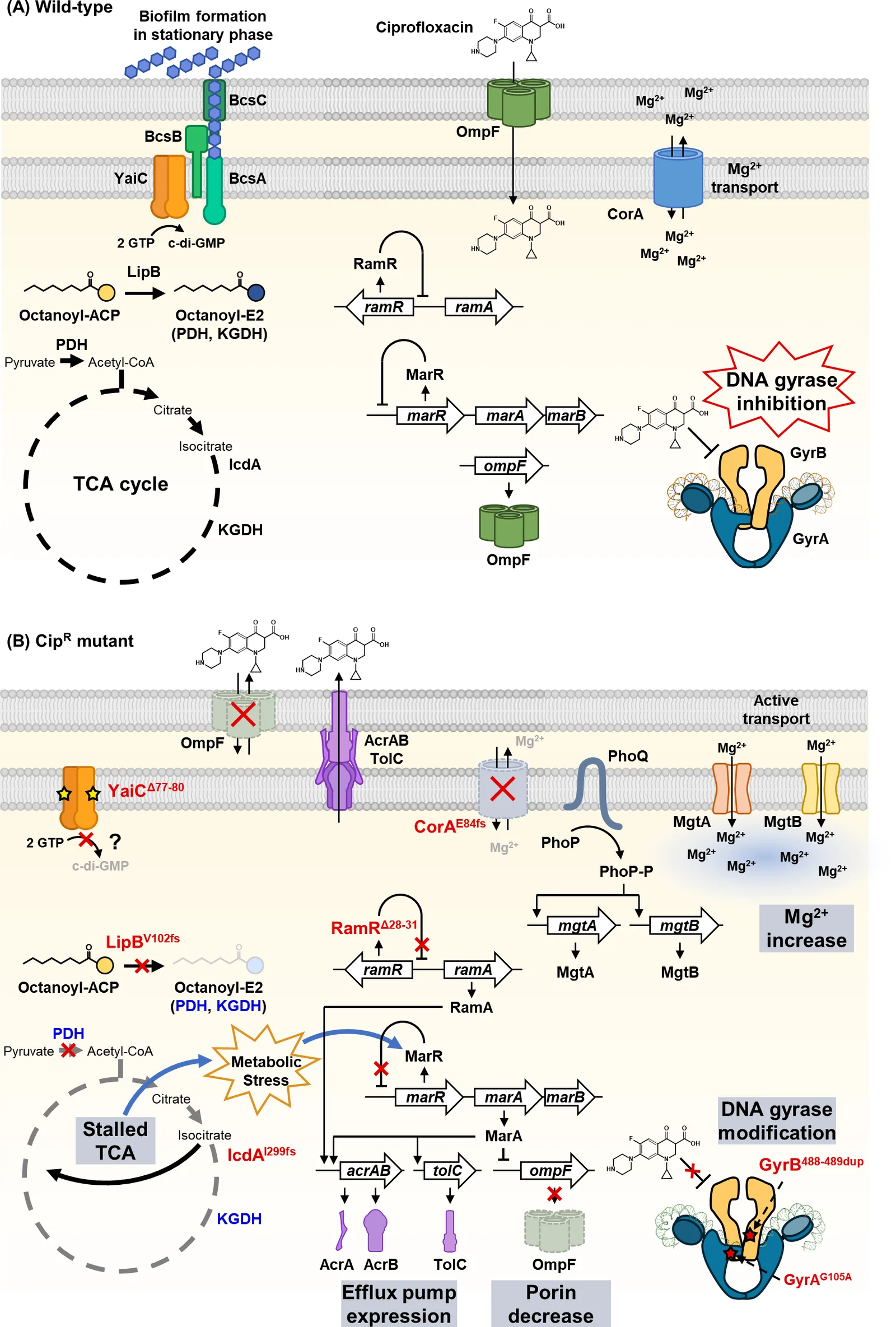
A proposed model of the mechanism of ciprofloxacin resistance in *Salmonella* Cip^R^ mutants. Overall responses to ciprofloxacin of the wild type (**A**) and Cip^R^ mutants (**B**). (**A**) In the wild type, ciprofloxacin enters through outer membrane porins such as OmpF and inhibits DNA gyrase, leading to bacterial death. (**B**) In contrast, Cip^R^ mutants acquire multiple chromosomal mutations that confer resistance. LipB^V102fs^ disrupts the function of the PDH and KGDH complexes, and IcdA^I299fs^ encodes a non-functional isocitrate dehydrogenase. Together, these mutations stall the TCA cycle and induce metabolic stress. This stress causes a conformational change in MarR, derepressing the *marRAB* operon and increasing *marA* expression. The *ramR*^Δ28–31^ mutation results in constitutive expression of RamA, which activates the *acrAB* operon. In parallel, MarA activates the expression of both AcrAB and TolC, thereby increasing the AcrAB-TolC efflux pump, enhancing ciprofloxacin efflux. At the same time, MarA represses the expression of the OmpF porin, reducing ciprofloxacin entry. The *gyrA*^G105A^ *gyrB*^488–489dup^ mutations alter the conformation of DNA gyrase, weakening its interaction with ciprofloxacin. Additionally, the *yaiC*^Δ77–80^ mutation abolishes c-di-GMP synthesis, thereby decreasing energy-consuming biofilm formation. The CorA^E84fs^ mutation leads to the production of a non-functional CorA Mg^2+^ transporter, probably increasing intracellular Mg^2+^ levels by failing to export Mg^2+^. Increased expression of alternative magnesium transporters *mgtA* and *mgtB* allowed the maintenance of high intracellular Mg^2+^ concentrations, which may stabilize the membrane and further decrease permeability.

In this study, we generated ciprofloxacin-resistant *Salmonella* mutants through continuous exposure to ciprofloxacin under laboratory conditions, following a method previously applied to generate ampicillin-resistant uropathogenic *E. coli* (32). Whole-genome sequencing identified four chromosomal coding mutations in *ramR*, *icdA*, *lipB,* and *gyrA* in the Low Cip^R^ mutant, and three additional mutations in *gyrB*, *yaiC*, and *corA* in the High Cip^R^ mutant. Structure and functional analyses revealed that, except for the essential genes *gyrA* and *gyrB*, all other mutated genes exhibited loss of functionality. Collectively, these mutations influenced ciprofloxacin resistance through increased efflux pump activity, disruption of the TCA cycle, and alterations in DNA gyrase activity (Fig. 10).

Efflux pumps are one of the most common mechanisms of antibiotic resistance, enabling bacteria to expel various antimicrobial agents and reduce their intracellular concentrations (16, 33). Mutations or deletions in *ramR* have been widely reported to contribute to multidrug resistance by increasing efflux pump activity. In *Salmonella*, specific *ramR* mutations such as T18P, Y59H, or M84I have been shown to decrease susceptibility to quinolones and other antibiotics (34, 35). In our Cip^R^ mutants carrying the *ramR*^Δ28–31^ mutation, the absence of the α2 helix in the DNA-binding domain was associated with significantly increased expression of *ramR*, *ramA,* and the efflux pump components *acrA* and *acrB* (Fig. 3D through G).

Furthermore, disruption of the TCA cycle causes metabolic slowdown and promotes the formation of persister cells (36). It also activates MarA, a global regulator that increases efflux pump activity and reduces OmpF porin expression, collectively lowering membrane permeability and enhancing antibiotic resistance (22–24). In our Cip^R^ mutants, the *icdA*^I299fs^ mutation disrupted isocitrate dehydrogenase, a key enzyme directly involved in the TCA cycle, while the *lipB*^V102fs^ mutation impaired octanoyl transferase, an essential enzyme required for the proper functioning of the PDH and KGDH complexes, leading to a stalled TCA cycle (Fig. 4F). Consequently, these alterations resulted in increased MarA expression and reduced OmpF expression (Fig. 4I and J), which likely limited ciprofloxacin influx and facilitated its efflux from the cell.

Mutations in the DNA gyrase subunits *gyrA* and *gyrB* are among the most critical mechanisms of fluoroquinolone resistance and are frequently observed in clinical isolates. These mutations are predominantly located in the winged-helix domain and the TOPRIM domain, which together constitute the central DNA gate of DNA gyrase (37–39) (Fig. S2 and S3). Previous studies have demonstrated that the Q106 residue in GyrA plays a key role in ciprofloxacin resistance (40). Given its proximity, we hypothesized that the G105A mutation might also contribute to resistance by altering the drug-binding site. However, our *in vitro* experimental data did not confirm the direct effect of this mutation on resistance. Because the substitution of glycine with alanine introduces only a small methyl (-CH_3_) group, this structural change is likely insufficient to significantly alter ciprofloxacin binding or disrupt DNA gyrase function. The High Cip^R^ mutant exhibited an additional GyrB^488–489dup^ mutation in the TOPRIM domain, a region critical for fluoroquinolone resistance (41). The duplication of two amino acids in this domain likely introduced structural alterations that impaired its functionality, thereby further reducing the efficacy of fluoroquinolone antibiotics. Consistent with this interpretation, *in vivo* plasmid supercoiling in the High Cip^R^ mutant was maintained even in the presence of ciprofloxacin (Fig. 5E), and the IC_50_ for moxifloxacin was higher than that of the wild-type strain (Fig. 5F and 5G; Fig. S3). Further structural studies will be required to define the underlying molecular mechanism. Notably, the mutations identified here differ from those most frequently reported in clinical and farm animal isolates of fluoroquinolone-resistant *Salmonella*, in which substitutions at GyrA Ser83 and Asp87 predominate (11, 26). The *ramR* deletion and TCA cycle-disrupting mutations in *icdA* and *lipB* identified in the Cip^R^ mutants are also uncommon in clinical isolates, where plasmid-mediated resistance mechanisms, such as *qnr* genes, are more prevalent (15). These differences suggest that resistance patterns arising under continuous laboratory selection pressure may diverge from those shaped by the complex selective environments encountered in clinical or agricultural settings.

The other mutations identified in the High Cip^R^ mutant, *yaiC* and *corA*, did not directly contribute to ciprofloxacin resistance, as evidenced by their inability to increase the MIC in deletion mutants (Fig. 9). However, these mutations may represent adaptive strategies that mitigate the physiological costs associated with ciprofloxacin resistance. Biofilm formation is generally recognized as a key mechanism that enhances bacterial resistance to antibiotics by acting as a physical barrier against drug penetration (42). Unexpectedly, the High Cip^R^ mutant acquired a mutation in *yaiC*, a gene essential for biofilm formation. Using a riboswitch-based sensor, we measured intracellular c-di-GMP levels and found that YaiC^Δ77–80^ was unable to synthesize c-di-GMP (Fig. 6E). Consistently, in the Congo red-based RDAR assay, YaiC^Δ77–80^ failed to form biofilm, further confirming its functional impairment (Fig. 6C). Notably, c-di-GMP levels were also lower than those of the wild-type strain in the Low Cip^R^ mutant, which lacks a *yaiC* lesion (Fig. 6F). Because *Salmonella* encodes numerous diguanylate cyclases (43), global c-di-GMP levels can be reduced even when the single gene remains intact. Given that TCA cycle disruption can constrain biofilm-associated metabolism (Fig. S1) (44), the *yaiC* lesion may reflect selection against an energetically costly trait that becomes less advantageous under continuous antibiotic pressure.

Magnesium stabilizes the bacterial envelope, reduces membrane permeability, and limits the influx of external compounds, including antibiotics (45). It also serves as an essential cofactor for various enzymes, such as DNA gyrase, supporting their structural integrity and activity (46, 47). In our study, the significant increase in *corA* and *mgtB* mRNA levels observed in the wild-type strain at high ciprofloxacin concentrations suggests a potential link between ciprofloxacin resistance and magnesium homeostasis (Fig. 7F and H). The emergence of the *corA*^E84fs^ mutation in the High Cip^R^ mutant suggests an adaptive shift in magnesium regulation under continuous antibiotic pressure. Although the precise mechanism remains unclear, the Low Cip^R^ mutant, which lacks the *corA* mutation, already exhibited elevated intracellular Mg^2+^ levels and increased *mgtA* and *mgtB* transcript levels even in the absence of ciprofloxacin (Fig. 7E, G, and H). By contrast, the High Cip^R^ mutant carrying *corA*^E84fs^ showed even higher *mgtA* and *mgtB* expression than the Low Cip^R^ mutant (Fig. 7G and H). Previous work has shown that loss of CorA triggers PhoP-independent MgtA production even under conditions of high extracellular Mg^2+^ (48).

As previous studies have shown that TCA cycle disruption enhances *Salmonella* survival in macrophages (49), it is possible that metabolic perturbations caused by TCA cycle disruptions may activate the PhoP/PhoQ two-component regulatory system, which requires further investigation.

One limitation of this study is that genomic mutations were analyzed from single resistant colonies rather than from the entire resistant population, which could provide a broader view of resistance-associated mutations. However, they lack the ability to isolate individual colonies for detailed physiological analyses. Further studies combining both approaches would be valuable for generating complementary insights into the mechanisms underlying ciprofloxacin resistance. Furthermore, whether the mutations identified under laboratory conditions are representative of those driving resistance in clinical or farm-derived isolates remains to be investigated.

## MATERIALS AND METHODS

### Bacterial strains, plasmids, oligodeoxynucleotides, and growth conditions

The bacterial strains and plasmids used in this study are listed in Table S1. All *S. enterica* serovar Typhimurium strains were derived from the wild-type strain 14028s. Bacteria were grown at 37°C in Luria-Bertani broth (MP Biomedicals, Cat. No. 3002032) and LB broth containing 0.2% glucose (Sigma-Aldrich, Cat. No. D9434) (50). For ciprofloxacin-resistant bacteria, 0.2 µg/mL ciprofloxacin was used in LB broth and solid medium with 0.2% glucose unless otherwise indicated. N-minimal medium supplemented with 0.1% casamino acids (Gibco, Cat. No. 223050), 38 mM glycerol (Sigma-Aldrich, Cat. No. G5516), and the indicated concentrations of MgCl_2_ (Biosesang, Cat. No. MR2001-001-00) was used (51). *Escherichia coli* DH5α was used as the host for preparing plasmid DNA. The ampicillin (Sigma-Aldrich, Cat. No. A0166) was used at 50 μg/mL, chloramphenicol (Biosesang, Cat. No. C1023) at 25 μg/mL, and kanamycin (Sigma-Aldrich, Cat. No. K1377) at 50 μg/mL. The ciprofloxacin (Sigma-Aldrich, Cat. No. 17850) was used at 0.12 or 0.5 µg/mL to measure antibiotic-induced mRNA expression.

### Isolation of ciprofloxacin-resistant strains

*S*. Typhimurium 14028s was cultured in LB broth medium. After 18 h of culture, cells were spread on LB agar medium containing 0.031 µg/mL ciprofloxacin. Plates were incubated for as short as 2–3 days or as long as 1–2 weeks until resistant colonies started to grow on the ciprofloxacin-containing plate. Resistant colonies were selected on LB agar medium containing 0.03 µg/mL ciprofloxacin and inoculated in LB broth medium with the same concentration of ciprofloxacin. This process was repeated by doubling the ciprofloxacin concentration up to 0.96 µg/mL (Low Cip^R^) or 1.5 µg/mL (High Cip^R^).

### Determination of minimal inhibitory concentration

The MIC was measured using the broth microdilution method (52). Briefly, the strains were cultured in Mueller-Hinton (MH) broth (Sigma-Aldrich, Cat. No. 70192). Various antibiotics, including ciprofloxacin, were diluted in MH broth to the 4× top concentration desired in the test. Using a multi-pipette, 50 μL of MH broth was dispensed into a 96-well plate, and 50 μL of diluted antibiotics was mixed into the wells of column 1. Half of the mixture was withdrawn and added to the next column. This procedure was repeated down to column 10, resulting in each well containing 50 μL of serially diluted antibiotics. Overnight-grown bacteria were diluted to the concentration suggested by the CLSI guidelines (about OD_600_ = 0.001), and 50 μL of the bacterial suspension was dispensed to the plate except column 11 (sterilized control). After incubating the plate overnight at 37°C, 30 μL of resazurin (0.015%, Sigma-Aldrich, Cat. No. 199303) was added to all wells and further incubated for 2–4 h for the observation of color change (53).

### Construction of bacterial strains

The one-step gene inactivation method (54) was used for chromosomal gene deletion. The sequences of primers are indicated in Table S1. For the construction of the *ramR, lipB, icdA,* and *yaiC* deletion strains, the Cm^R^ cassette from plasmid pKD3 (for *ramR*, *icdA*, and *yaiC*) and the Km^R^ cassette from plasmid pKD4 (for *lipB*) were amplified using appropriate primers, and the resulting PCR products were integrated into the chromosome to generate *ramR*::Cm^R^, *icdA*::Cm^R^, *yaiC*::Cm^R^, and *lipB*::Km^R^. From these deletion mutants, the antibiotic cassettes were removed using plasmid pCP20 (54). A P22 phage lysate grown in strain *icdA*::Cm^R^ transduced the Δ*lipB* strain for generating the TCA stalled strain (Δ*icdA* Δ*lipB*). The deletion of the corresponding genes was verified by sequencing.

### Construction of plasmids

For the analyses of the red-dry-rough phenotype assay, the plasmids pBAD33-*yaiC* and *yaiC*^ΔF77–P80^ were constructed as follows: PCR with the primer pairs NJP257/NJP258 using 14028s (for *yaiC*) and EN1751 (for *yaiC*^ΔF77–P80^) colony as templates, respectively. The amplified DNA fragments were digested with KpnI and XbaI and cloned into pBAD33 digested with the same enzymes. The sequence of the resulting constructs was verified by DNA sequencing. For c-di-GMP measurement, the plasmid pCDGRS was constructed by inserting a c-di-GMP riboswitch sequence, designed based on a previously reported riboswitch (55), upstream of the *gfp* gene. The c-di-GMP riboswitch oligonucleotide with HindIII and XbaI restriction sites was synthesized by Macrogen (Korea). The backbone plasmid pFCcGi (Addgene, Cat. No. 59324) was digested with HindIII and XbaI, and the riboswitch sequence was ligated into the digested site.

### Western blot analysis

Cells were grown for 4 h 30 min in 10 mL (wild type) or 20 mL (Low Cip^R^, High Cip^R^, and Δ*lipB*) LB containing 0.2% glucose without (−) or with (+) ciprofloxacin. Cells were normalized by measuring the OD_600_. Crude extracts were prepared in Tris-buffered saline (Biosesang, Cat. No. TR2008-100-00) by sonication. Whole-cell lysates were resolved on 12% SDS-polyacrylamide gels with a protein marker (Bio-Rad, Cat. No. 1610374) and were transferred onto nitrocellulose membranes. The blot was incubated overnight with polyclonal anti-lipoic acid antibody (1:5,000 dilution; Abcam, catalog no. Ab58724) to detect lipoic acid. The blot was developed by incubation with anti-rabbit IgG horseradish peroxidase-linked antibody (Ab) (1:10,000 dilution; ThermoFisher, catalog no. 31460) for 1 h and visualized using SuperSignal West Femto maximum-sensitivity substrate (Thermo Fisher, Cat. No. 34096).

### Real-time quantitative reverse transcription PCR

Total RNA was isolated using the RNeasy kit (Qiagen, Cat. No. 74104) according to the manufacturer’s instructions. The purified RNA was quantified using a NanoDrop machine (ThermoFisher). cDNA was synthesized using a PrimeScript RT reagent kit (TaKaRa, Cat. No. RR037A-6). Transcripts were quantified by real-time PCR using the SYBR Green PCR master mix (Applied Biosystems) on a StepOnePlus real-time PCR system (Applied Biosystems, Cat. No. A25742). The primers used for the detection of transcripts of each gene are listed in Table S1. The mRNA levels of each target gene were calculated using a standard curve of *Salmonella* 14028s genomic DNA at known concentrations, and the data were normalized to 16S rRNA levels of the *rrsH* gene. All real-time qRT-PCR results were derived from three biological replicates.

### DNA supercoiling assay

To assess the DNA supercoiling levels of Cip^R^ mutants, the pBR322 plasmid was transformed into wild-type and Cip^R^ strains. After incubation in LB medium supplemented with 0.2% glucose for 1.5 h, ciprofloxacin was added to a final concentration of 1 μg/mL, and the cultures were further incubated for 1 h. Cells were harvested, and plasmids were extracted using the ZymoPURE Plasmid Miniprep Kit (Zymo Research, Cat. No. D4209). Equal amounts of plasmid were loaded onto a 1.5% agarose gel with a DNA ladder (TransGen, Cat. No. BM211) and separated by electrophoresis. The DNA states were quantified using ImageJ software, and the degree of DNA supercoiling was calculated as the supercoiled-to-linear ratio.

### DNA gyrase purification

*Salmonella* GyrA(1–861) and GyrB(6–804) encoding sequences were cloned into pET30b (69910, Sigma-Aldrich) using NdeI and XhoI and into the pET-HIS vector (56) using BamHI and HindIII, respectively. The constructs were transformed into *E. coli* BL21/De3 ER2566 (D1039S, Beyotime, Beijing) and cultured until the OD_600_ reached 0.6–0.8. The target proteins were expressed by induction with 0.4 mM isopropyl β-D-1-thiogalactopyranoside (1758-1400, Inalco, USA). GyrA(1–861) was induced at 37°C for 4 h, while GyrB(6–804) was induced at 18°C for 16 h. Cells were harvested and resuspended in a lysis buffer of 20 mM Tris (pH 8.0), 500 mM NaCl, 20 mM imidazole, 5 mM β-mercaptoethanol, and 10% (vol/vol) glycerol. The cells were lysed using sonication in an ultrasonicator (SCIENTZ-IID, Ningbo, China). The lysis supernatants after 18,000 rpm centrifugation at 4°C were collected and first purified with 1 mL nickel affinity agarose (SA004100, Smart, Changzhou, China). The best fractions of proteins were collected and further purified by Superdex S200 (28990944, Cytiva, USA). Purified proteins were concentrated in a buffer of 20 mM Tris (pH 8.0), 50 mM monosodium glutamate, 50 mM potassium acetate, 0.5 mM DTT, 1 mM EDTA, and 10% glycerol, snap frozen in liquid nitrogen, and stored at −80°C. All restriction enzymes were purchased from New England Biolabs.

### Cleaved complex assay

GyrA_2_B_2_ enzyme was assembled using equimolar amounts of GyrA and GyrB proteins in their storage buffer. The reaction was set up with a final concentration of 50 nM enzyme, 1 mM ATP, and pET-HIS plasmid DNA as its substrate in a reaction buffer of 25 mM Tris (pH 7.5), 25 mM KCl, 5 mM MgCl_2_, 5 mM DTT, 1 mM spermidine, 100 μg/mL BSA, and 5% glycerol. The inhibitor moxifloxacin (BD32396, Bidepharm, Shanghai) with concentrations ranging from 0.02 to 327.68 μg/mL was prepared in the reaction buffer. The reaction mixture was incubated at 37°C for 30 min and further mixed with a 3× loading buffer of 0.13% (wt/vol) bromophenol blue, 15% (wt/vol) glycerol, 3% SDS, 30 mM EDTA, and 0.1% Gel Red (MK02, Magen, Guangzhou, China). A 0.8% agarose gel was run at 50 V for 60 min to separate different plasmid conformations. A 1 kb DNA ladder (BM201-01, TransGen, Beijing) was used as a size marker. The gels were imaged using a Sangon Blue Gel Imaging System. The band intensities of supercoiled and relaxed/linear DNA were quantified using ImageJ software (NIH). Dose-dependent responses were fitted by a nonlinear regression model in Excel. The inhibition rate (IC_50_) was calculated based on the reduction of supercoiled DNA.

### Red, dry, and rough assay

RDAR assays were performed using wild type, Cip^R^ mutants, and Δ*yaiC* mutant and their complemented strains to evaluate biofilm formation. The wild type and Cip^R^ mutants were spotted onto LB agar plates without NaCl and supplemented with 40 μg/mL Congo red (Sigma-Aldrich, Cat. No. 234610) and 20 μg/mL Coomassie brilliant blue (Sigma-Aldrich, Cat. No. B0770) (29). For complementation analysis, wild type/pBAD33, Δ*yaiC*/pBAD33, Δ*yaiC*/pBAD33-*yaiC*, and Δ*yaiC*/pBAD33-*yaiC*^ΔF77–P80^ were spotted onto plates further supplemented with 1 mM arabinose (Sigma-Aldrich, Cat. No. A3256) and 25 μg/mL chloramphenicol. All plates were incubated at room temperature for 4–6 days.

### Measuring intracellular c-di-GMP levels

To assess intracellular c-di-GMP levels, the plasmid pCDGRS containing a c-di-GMP riboswitch was transformed into wild-type/pBAD33, Δ*yaiC*/pBAD33, Δ*yaiC*/pBAD33-*yaiC*, and Δ*yaiC*/pBAD33-*yaiC*^ΔF77–P80^ strains used for complementation analysis. Strains were precultured in LB medium supplemented with 100 μg/mL ampicillin and 25 μg/mL chloramphenicol and then diluted 1:100 into the same medium for main culture. To induce YaiC or YaiC^ΔF77–P80^ expression, 1 mM arabinose was added at the beginning of the main culture. After 5 h of incubation, GFP fluorescence (excitation, 467 nm; emission, 510 nm) and mCherry fluorescence (excitation, 585 nm; emission, 620 nm) were measured using a Synergy H1 plate reader (BioTek, Korea). GFP intensity was normalized to mCherry fluorescence to determine the relative c-di-GMP levels.

To evaluate c-di-GMP levels in response to ciprofloxacin, the pCDGRS plasmid was transformed into wild-type, Low Cip^R^, and High Cip^R^ strains. Strains were grown in LB medium supplemented with 100 μg/mL ampicillin and ciprofloxacin at final concentrations of 0, 0.125, or 0.5 μg/mL. After 5 h of incubation, GFP and mCherry fluorescence were measured as described above, and GFP fluorescence was normalized to mCherry to assess the relative c-di-GMP levels.

### β-galactosidase assay

β-galactosidase activity was determined as previously described (30). Overnight cultures were grown in N-minimal medium containing 10 mM Mg^2+^. After washing twice with Mg^2+^-free medium, cells were grown for 6 or 24 h in N-minimal medium containing either 10 mM or 10 µM Mg^2+^. Aliquots (200 µL) of each culture were transferred to 1.5 mL tubes, followed by the addition of chloroform (Sigma-Aldrich, Cat. No. 372978) and SDS (Biosesang, Cat. No. SR2003-050-00). Samples were then vortexed for 30 s and incubated at room temperature for 20 min to allow debris to settle. Seventy-five microliters of the clarified supernatant was transferred to a 96-well plate and mixed with an equal volume of ONPG (Sigma-Aldrich, Cat. No. 73660) prepared in Z-buffer (Biosolution, Cat. No. BZ001). β-galactosidase activity was quantified using a Synergy H1 plate reader (BioTek).

### Intramacrophage survival assay

Intramacrophage survival assay was conducted with the macrophage-like cell line J774A.1 as described (31). Briefly, 3 × 10^5^ macrophages in Dulbecco’s modified Eagle’s medium (DMEM, Welgene, Cat. No. LM001-05) supplemented with 10% fetal bovine serum (FBS, Welgene, Cat. No. S001-07) were seeded in 24-well plates and cultured at 37°C. Overnight-grown bacteria were added to the macrophages at a multiplicity of infection of 20, and the plates were centrifuged at 1,500 rpm for 5 min at room temperature and incubated for an additional 30 min. For measuring the number of bacteria at *T*_0_, cells were lysed with phosphate-buffered saline (PBS, Welgene, Cat. No. LB001-02) containing 0.1% Triton X-100 (Sigma-Aldrich, Cat. No. T8787) and plated on Luria-Bertani broth plates with appropriate dilutions. Then, the extracellular bacteria were washed three times with PBS and killed by incubation with DMEM supplemented with 10% FBS containing 150 μg/mL gentamicin (Sigma-Aldrich, Cat. No. G1397) and 1 µg/mL ciprofloxacin for 1 h. For measuring the number of bacteria at 1 h postinfection, cells were lysed with PBS containing 0.1% Triton X-100 and plated on Luria-Bertani broth plates with appropriate dilutions. Finally, for measuring the number of bacteria at 21 h, the DMEM was replaced after 1 h with fresh DMEM containing 15 μg/mL gentamicin and 1 µg/mL ciprofloxacin, and the incubation was continued at 37°C. After 21 h, cells were lysed with PBS containing 0.1% Triton X-100 and plated on Luria-Bertani broth plates. The fold replication within macrophages was calculated by dividing the colony count at *T*_21_ by the colony count at *T*_1_. The fold invasion was determined by dividing the colony count at *T*_1_ by the number of infected bacteria, and the fold attachment was calculated by dividing the colony count at *T*_0_ by the number of infected bacteria.

### Whole-genome sequencing

The whole-genome sequencing was performed as described (32). Briefly, genomic DNA from each strain was extracted using a G-spin Genomic DNA Extraction Kit (iNtRON, Cat. No. 17121). Sixty micrograms per microliter of genomic DNA was processed to construct a sequencing library using a TruSeq Nano DNA Sample Prep Kit (Illumina, USA, #20015964). The sequencing was performed using the NovaSeq 6000 platform (Illumina, USA) on a 151 bp paired-end platform. We obtained an average of 5.62 Gb of data (BioProject accession number PRJNA1398555). Raw sequencing reads generated from three strains, including the control strain, were subjected to adapter removal and quality trimming using Trimmomatic v0.36 (57). The resulting cleaned reads were mapped to the reference genome, *S*. Typhimurium 14028s (PRJNA224116), using CLC Genomics Workbench 21 (Qiagen). The reads showed an average mapping rate of 99.94%, providing a mean sequencing depth of more than 1,000× for each sample. Variant calling was performed using the Basic Variant Detection tool implemented in CLC Genomics Workbench 21, and identified variants were subsequently compared among the strains.

### Structural modeling

All three-dimensional protein structures were predicted using AlphaFold 3 (58) via the AlphaFold Server (https://alphafoldserver.com). All structural figures were generated using PyMOL (Schrödinger, LLC).

## Data Availability

The data that support the findings of this study are available in the NCBI SRA database at https://www.ncbi.nlm.nih.gov/bioproject/PRJNA1398555. All other relevant data are publicly available in Figshare at https://doi.org/10.6084/m9.figshare.31249513 (59).

## SUPPLEMENTAL MATERIAL

Supplemental figures and table spectrum.01194-26-s0001.pdf Figures S1 to S3 and Table S1.

ASM does not own the copyrights to Supplemental Material that may be linked to, or accessed through, an article. The authors have granted ASM a non-exclusive, world-wide license to publish the Supplemental Material files. Please contact the corresponding author directly for reuse.
