# Supplementary material for: Genetic mutations driving ciprofloxacin resistance in laboratory-evolved Salmonella Typhimurium
Source: Microbiol Spectr. 2026 Sep 8;14(10):e01194-26. doi: 10.1128/spectrum.01194-26 (PMC13689460; doi:10.1128/spectrum.01194-26)
Supplement: Supplementary file 1 [file spectrum.01194-26-s0001.pdf]

Supplementary Information

Genetic mutations driving ciprofloxacin resistance in laboratory-evolved *Salmonella* Typhimurium

Nakjun Choi [a] *, Eunna Choi [a] *, Yong-Joon Cho [b], Yajing Wen [c], Jiangban Zhao [c], Aidong Han [c] #, and Eun-Jin Lee [a] #

[a] Department of Life Sciences, School of Life Sciences and Biotechnology, Korea University, Seoul, South Korea

[b] Department of Molecular Bioscience, Multidimensional Genomics Research Center, College of Biomedical Science, Kangwon National University, Chuncheon, South Korea

[c] Department of Biochemistry and Molecular Biology, School of Life Sciences, Xiamen University, Xiang'an, Xiamen 361102, China

Supplementary Information includes

Figures S1-S3

Table S1

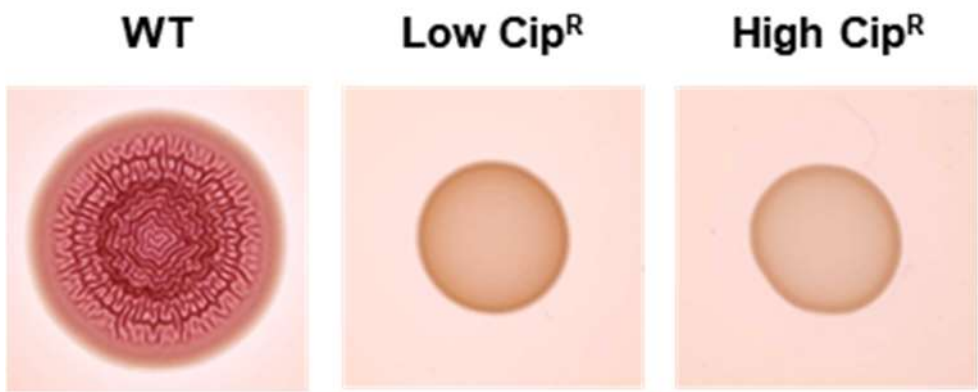

Figure S1. RDAR assay

The RDAR assay of wild-type and Cip^R mutants. Strains were spotted on LB without NaCl containing Congo red and Coomassie blue and incubated at room temperature for 4 days.

(A)

GyrA

WHD

Tower

Coiled-coil

β-pinwheel

```
GYRA_ECOLI      MSDLAREITPVNIEEELKSSYLDYAMSVIVGRALPDVRDGLKPVHRRVLYAMNVLGNDWN
GYRA_SALTY      MSDLAREITPVNIEEELKSSYLDYAMSVIVGRALPDVRDGLKPVHRRVLYAMNVLGNDWN
                ************************************************************

GYRA_ECOLI      KAYKKSARVVGDVIGKYHPHGDSAVYDTIVRMAQPFSLRYMLVDGQGNFGSIDGDSAAAM
GYRA_SALTY      KAYKKSARVVGDVIGKYHPHGDSAVYDTIVRMAQPFSLRYMLVDGQGNFGSIDGDSAAAM
                ************************************************************

GYRA_ECOLI      RYTEIRLAKIAHELMADLEKETVDFVDNYDGTEKIPDVMPTKIPNLLVNGSSGIAVGMAT
GYRA_SALTY      RYTEIRLAKIAHELMADLEKETVDFVDNYDGTEKIPDVMPTKIPNLLVNGSSGIAVGMAT
                ************************************************************

GYRA_ECOLI      NIPPHNLTEVINGCLAYIDDEDISIEGLMEHIPGPDFPTAAIINGRRGIEEAYRTGRGKV
GYRA_SALTY      NIPPHNLTEVINGCLAYIDNEDISIEGLMEHIPGPDFPTAAIINGRRGIEEAYRTGRGKV
                *******************:****************************************

GYRA_ECOLI      YIRARAEVEVDAKTGRETIIVHEIPYQVNKARLIEKIAELVKEKRVEGISALRDESDKDG
GYRA_SALTY      YIRARAEVEADAKTGRETIIVHEIPYQVNKARLIEKIAELVKDKRVEGISALRDESDKDG
                *********.********************************:*****************

GYRA_ECOLI      MRIVIEVKRDAVGEVVLNNLYSQTQLQVSFGINMVALHHGQPKIMNLKDIIAAFVRHRRE
GYRA_SALTY      MRIVIEVKRDAVGEVVLNNLYSQTQLQVSFGINMVALHHGQPKIMNLKDIISAFVRHRRE
                ***************************************************:********

GYRA_ECOLI      VVTRRTIFELRKARDRAHILEALAVALANIDPIIELIRHAPTPAEAKTALVANPWQLGNV
GYRA_SALTY      VVTRRTIFELRKARDRAHILEALAIALANIDPIIELIRRAPTPAEAKAALISRPWDLGNV
                ************************:*************:********:**::.**:****

GYRA_ECOLI      AAMLERAGDDAARPEWLEPEFGVRDGLYYLTEQQAQAILDLRLQKLTGLEHEKLLDEYKE
GYRA_SALTY      AAMLERAGDDAARPEWLEPEFGVRDGQYYLTEQQAQAILDLRLQKLTGLEHEKLLDEYKE
                ************************** *********************************

GYRA_ECOLI      LLDQIAELLRILGSADRLMEVIREELELVREQFGDKRRTEITANSADINLEDLITQEDVV
GYRA_SALTY      LLEQIAELLHILGSADRLMEVIREEMELIRDQFGDERRTEITANSADINIEDLISQEDVV
                **:******:***************:**:*:****:*************:****:*****

GYRA_ECOLI      VTLSHQGYVKYQPLSEYEAQRRGGKGKSAARIKEEDFIDRLLVANTHDHILCFSSRGRVY
GYRA_SALTY      VTLSHQGYVKYQPLTDYEAQRRGGKGKSAARIKEEDFIDRLLVANTHDTILCFSSRGRLY
                **************::******************************** *********:*

GYRA_ECOLI      SMKVYQLPEATRGARGRPIVNLLPLEQDERITAILPVTEFEEGVKVFMATANGTVKKTVL
GYRA_SALTY      WMKVYQLPEASRGARGRPIVNLLPLEANERITAILPVREYEEGVNVFMATASGTVKKTAL
                 *********:*************** :********* *:****:******.******.*

GYRA_ECOLI      TEFNRLRTAGKVAIKLVDGDELIGVDLTSGEDEVMLFSAEGKVVRFKESSVRAMGCNTTG
GYRA_SALTY      TEFSRPRSAGIIAVNLNDGDELIGVDLTSGSDEVMLFSAAGKVVRFKEDAVRAMGRTATG
                ***.* *:** :*::* *************.******** ********.:***** .:**

GYRA_ECOLI      VRGIRLGEGDKVVSLIVPRGDGAILTATQNGYGKRTAVAEYPTKSRATKGVISIKVTERN
GYRA_SALTY      VRGIKLAGDDKVVSLIIPRGEGAILTVTQNGYGKRTAADEYPTKSRATQGVISIKVTERN
                ****:*. .*******:***:*****.**********. *********:***********

GYRA_ECOLI      GLVVGAVQVDDCDQIMMITDAGTLVRTRVSEISIVGRNTQGVILIRTAEDENVVGLQRVA
GYRA_SALTY      GSVVGAVQVDDCDQIMMITDAGTLVRTRVSEISVVGRNTQGVILIRTAEDENVVGLQRVA
                * *******************************:**************************

GYRA_ECOLI      EPVDEEDLDTIDGSAAEGDDEIAPEVDVDDEPEEE---
GYRA_SALTY      EPVDDEELDAIDGSVAEGDEDIAPEAESDDDVADDADE
                ****:*:**:****.****::****.: **:  ::
```

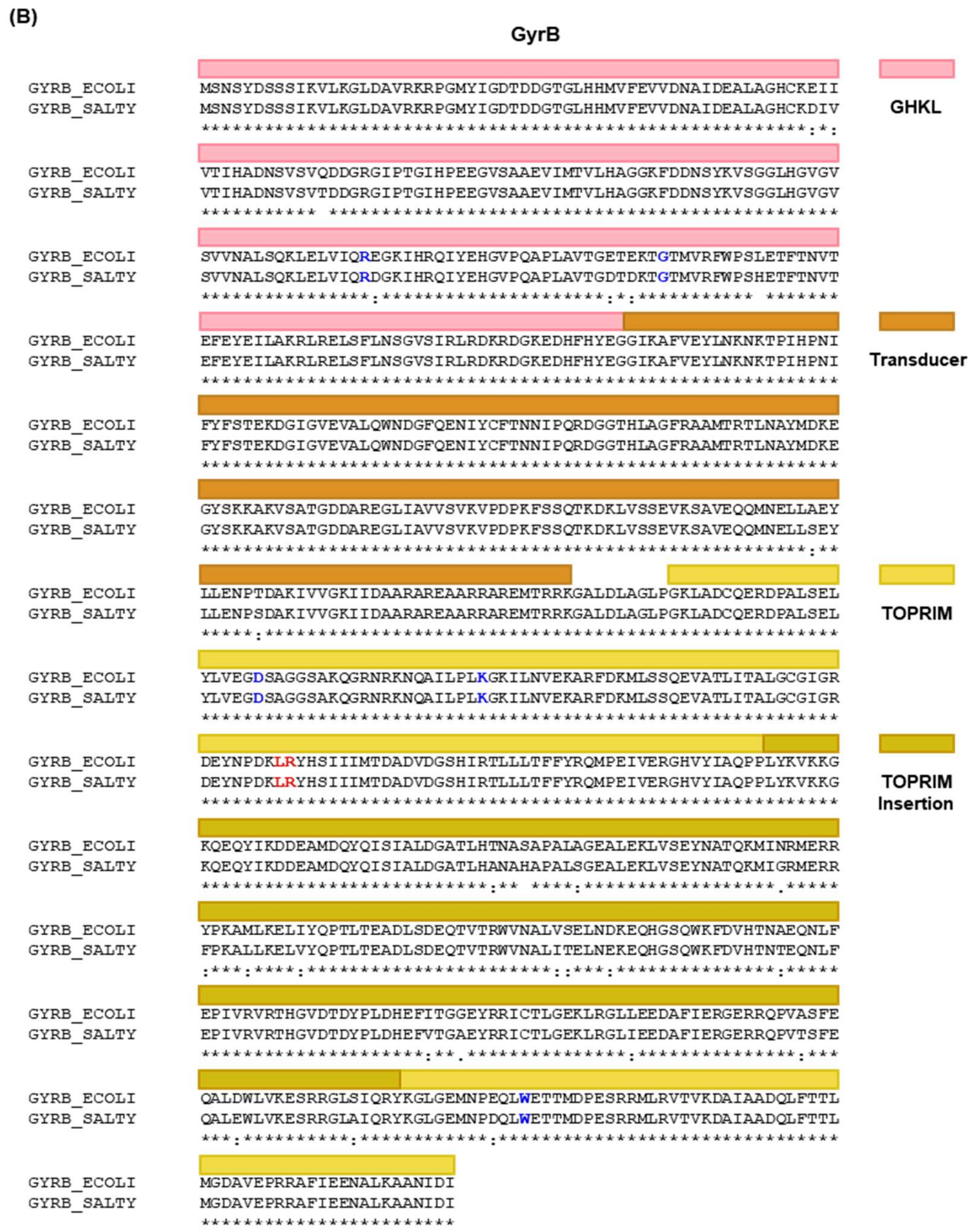

Figure S2. Domain structure of DNA gyrase and fluoroquinolone resistance–associated mutation sites

Schematic representation of DNA gyrase subunits aligned between *Escherichia coli* and *Salmonella* Typhimurium. Mutation sites known to be associated with fluoroquinolone resistance are shown in blue, and the mutations identified in our Cip^R mutants are shown in red.

(A) GyrA subunit. Most resistance-associated mutations are located in the WHD domain.

(B) GyrB subunit. Most resistance-associated mutations are located in the TOPRIM domain.

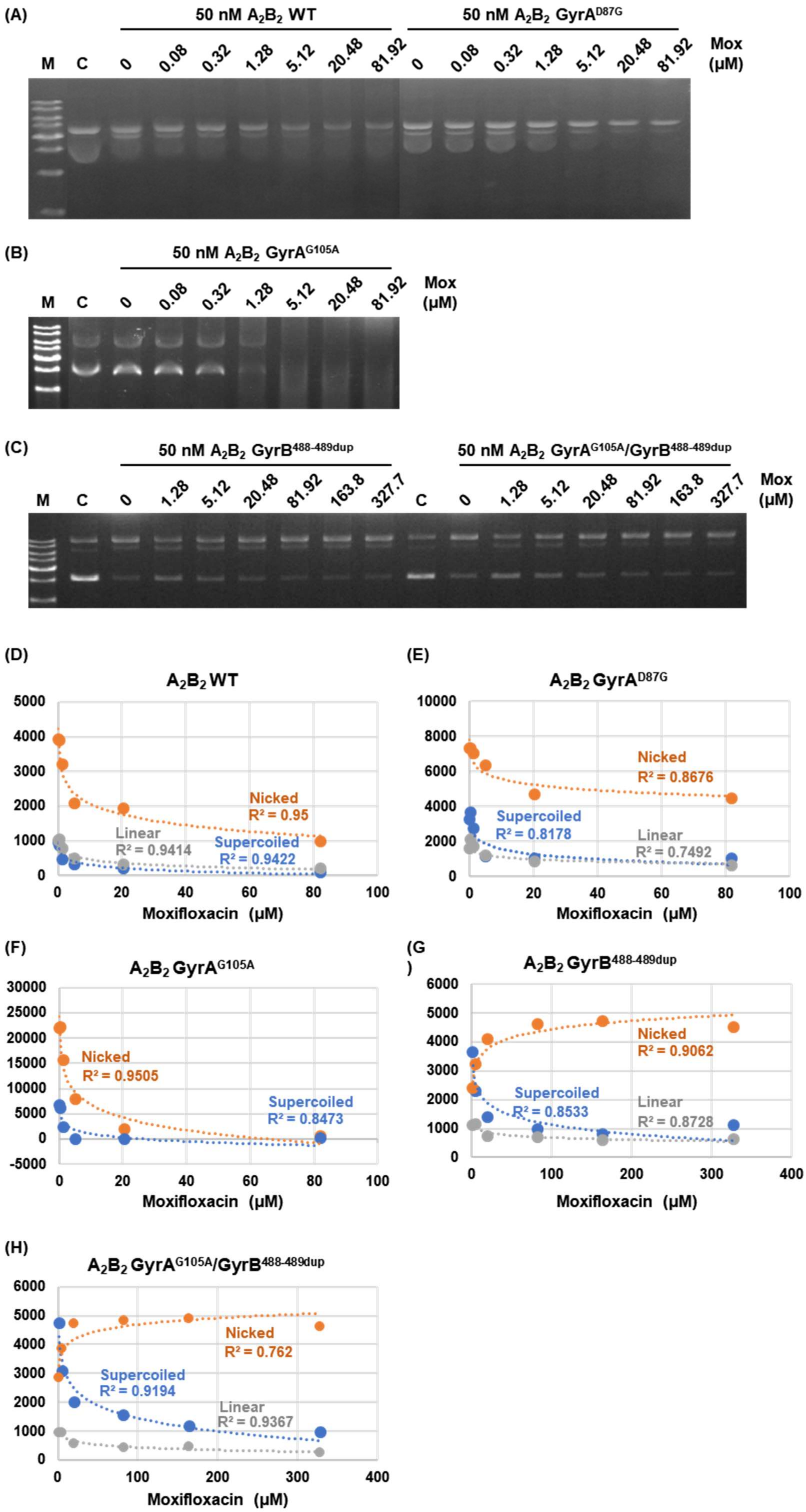

(A)
50 nM A2B2 WT
50 nM A2B2 GyrAD87G
M C 0 0.08 0.32 1.28 5.12 20.48 81.92 0 0.08 0.32 1.28 5.12 20.48 81.92
Mox (µM)
(B)
50 nM A2B2 GyrAG105A
M C 0 0.08 0.32 1.28 5.12 20.48 81.92
Mox (µM)
(C)
50 nM A2B2 GyrB488-489dup
50 nM A2B2 GyrAG105A/GyrB488-489dup
M C 0 1.28 5.12 20.48 81.92 163.8 327.7 C 0 1.28 5.12 20.48 81.92 163.8 327.7
Mox (µM)
(D)
A2B2 WT
Nicked
R² = 0.95
Linear
R² = 0.9414
Supercoiled
R² = 0.9422
Moxifloxacin (µM)
(E)
A2B2 GyrAD87G
Nicked
R² = 0.8676
Supercoiled
R² = 0.8178
Linear
R² = 0.7492
Moxifloxacin (µM)
(F)
A2B2 GyrAG105A
Nicked
R² = 0.9505
Supercoiled
R² = 0.8473
Moxifloxacin (µM)
(G)
A2B2 GyrB488-489dup
Nicked
R² = 0.9062
Supercoiled
R² = 0.8533
Linear
R² = 0.8728
Moxifloxacin (µM)
(H)
A2B2 GyrAG105A/GyrB488-489dup
Nicked
R² = 0.762
Supercoiled
R² = 0.9194
Linear
R² = 0.9367
Moxifloxacin (µM)

Figure S3. Moxifloxacin IC_{50} determination for reconstituted DNA gyrase variants
(A–C) Representative agarose gels of gyrase-mediated supercoiling reactions performed with relaxed plasmid DNA in the presence of increasing concentrations of moxifloxacin. Heterotetrameric $GyrA_2B_2$ (50 nM) was assembled from purified GyrA and GyrB subunits and incubated with 1 mM ATP in reaction buffer. Bands corresponding to supercoiled, nicked, and linear DNA are indicated. (D–H) Quantification of supercoiled (blue), nicked (orange), and linear (gray) fractions across the moxifloxacin series for the indicated gyrase variants. Band intensities were measured using ImageJ, and dose–response curves were fit by nonlinear regression. IC_{50} values were defined from the supercoiled fraction as the drug concentration that reduced that fraction to 50% of the no-drug level.

Table S1. Bacterial strains, plasmids, and oligonucleotides were used in this study.

(A) **Bacterial strains and plasmids**

Name	Description	Reference
***S. enterica* serovar Typhimurium strains**		
14028s	Wild-type	(1)
EN1749	Low Cip^R	This study
EN1751	High Cip^R	This study
EN1792	Δ*yaiC*::Cm	This study
NJ294	Δ*yaiC*	This study
EN1797	Δ*lipB*::Km	This study
NJ293	Δ*lipB*	This study
NJ292	Δ*icdA*	This study
EN1805	Δ*ramR*::Cm	This study
EN1804	Δ*lipB*, Δ*icdA*::Cm	This study
EN473	Δ*corA*	(2)
EN1786	Δ*mgtB*, *mgtA*::MudJ	This study
EN1789	14028s / pBR322	This study
EN1790	Low Cip^R / pBR322	This study
EN1791	High Cip^R / pBR322	This study
NJ214	14028s / pBAD33	This study
NJ295	Δ*yaiC* / pBAD33	This study
NJ296	Δ*yaiC* / pBAD33-*yaiC*	This study
NJ297	Δ*yaiC* / pBAD33-$yaiC^{\Delta F77\ to\ P80}$	This study
NJ316	14028s / pBAD33, pCDGRS	This study
NJ317	Δ*yaiC* / pBAD33, pCDGRS	This study
NJ318	Δ*yaiC* / pBAD33-*yaiC*, pCDGRS	This study
NJ319	Δ*yaiC* / pBAD33-$yaiC^{\Delta F77\ to\ P80}$, pCDGRS	This study
EL399	Wild-type / pYS1010	(3)
EN1701	Δ*corA* / pYS1010	This study
EN1702	Low Cip^R / pYS1010	This study
EN1703	High Cip^R / pYS1010	This study
**Plasmids**		
pCP20	rep_{pSC101}^{ts} Ap^R Cm^R *FLP*$^+$ *c*I857$^+$	(4)
pKD3	$repR_{R6K}$ Ap^R FRT Cm^R FRT	(4)
pKD4	$repR_{R6K}$ Ap^R FRT Km^R FRT	(4)
pKD46	rep_{pSC101}^{ts} Ap^R p_{araBAD} exo	(4)
NJ306	pBAD33-*yaiC*	This study
NJ307	pBAD33-$yaiC^{\Delta F77\ to\ P80}$	This study
pYS1010	rep_{p15A} Cm^R $p_{lac1\text{-}6}$ lacZ $_{this}$ *mgtA* 5'$UTR_{1\text{-}264}$	(3)
pUC57-Simple_CDGRS	rep_{pUC} Ap^R c-di-GMP riboswitch	This study
pCDGRS	oriRSF1010 Ap^R constitutive::mCherry Pbad::c-di-GMP riboswitch-EGFP	This study

(B) Primers used in this study

Name	Sequence (5' to 3')	
**For deletion mutant strains**		
KU880	TGATTCAATCATGTTTTTCCAGACATAAGGGTAACGTGTATGTAGG CTGGAGCTGCTTCG	Del-*ramR*-F
KU881	GCGCGCCACATGGCTTCGAATCCCAGCGCAATATATTCGCCATATG AATATCCTCCTTAG	Del-*ramR*-R
KU888	GGTTGACATTCACCGTTGCGAC	Del-*ramR*-con-F
KU889	GCCAGCAGTGTTCGGTAAAAGG	Del-*ramR*-con-R
KU882	CCGATATGCGCGATGAAAACAGCCATGATGAAATCTGGTTTGTAGG CTGGAGCTGCTTCG	Del-*lipB*-F
KU883	TAATGACCCATAAATTTCTTGGGCCATTATATTTACGCACATATGAAT ATCCTCCTTAG	Del-*lipB*-R
KU890	CGGAGATGCCGTTTTGTATCA	Del-*lipB*-con-F
KU891	CATGAGGACTGAAGAGGCCAT	Del-*lipB*-con-R
KU884	TGACAGACGAGCAAACCAGAAGCGCTCGAAGGAGAGGTGATGTA GGCTGGAGCTGCTTCG	Del-*icdA*-F
KU885	GAACCTTCGGCTCCCGTTCTTGTTTAACCCAAAAACTGGACATATGA ATATCCTCCTTAG	Del-*icdA*-R
KU892	ATTGCTCTAACGCAGTCGTGC	Del-*icdA*-con-F
KU893	GAAATCATAAGGGTTTTGGGG	Del-*icdA*-con-R
KU886	CATGCGCTCTGTTTCTATAATTTGGGAAAATTGTTTCTAATGTAGG CTGGAGCTGCTTCG	Del-*yaiC*-F
KU887	TGCCGCCACTTCGGTGCGGTTACGTCCGGCATTCTTTGCTACATAT GAATATCCTCCTTAG	Del-*yaiC*-R
KU894	GCTATCGGGTGAGTCCTGAAA	Del-*yaiC*-con-F
KU895	GCGATGACGAAGTGTATGGAAA	Del-*yaiC*-con-R
**For plasmid**		
NJP257	TCAGGTACCTCACCCGAAATTTCATACTTCCTCCATGC	*yaiC*-KpnI-F
NJP258	CTGTCTAGAGCCCGGCTGGACGTCATG	*yaiC*-XbaI-R
NJP261	CTGAAGCTTGGTTTTCCCAGTCACGACGTTG	CDGRS_HindIII_F
NJP262	GTGTCTAGAGCGGATAACAATTTCACACAGGAAAC	CDGRS_XbaI_R
AHP001	GACCATATGAGCGACCTTGCGAGA	GyrA-n1Nde
AHP002	GACCTCGAGATCCTCATCCCCTTCCG	GyrA-c861Xho
AHP003	GACGGATCCGACTCCTCCAGTATCAAAG	GyrB-n6Bam
AHP004	GACAAGCTTTTAAATATCGATATTCGCCG	GyrB-c804Hind
AHP005	GTGTATGGCACCATCGTTCG	GyrA-D87G1
AHP006	ATGGTGCCATACACTGCGG	GyrA-D87G2
AHP007	GTGGATGCTCAGGGTAACTTCG	GyrA-G105A1
AHP008	CTGAGCATCCACCAGCATG	GyrA-G105A2
AHP009	AAGCTGCGCCTGCGCTATCAC	GyrB-488i1
AHP010	ATAGCGCAGGCGCAGCTTGTC	GyrB-488i2
**For qRT PCR**		
KHQ163	TGGCCGTCAGCGAAAAG	*ramR* qRT PCR-F
KHQ164	AACACCATCAAAACGGAACGA	*ramR* qRT PCR-R
KHQ153	TTTGAATCAGCCGTTACGTATTGA	*ramA* qRT PCR-F
KHQ154	CAGACTCTCCCCTTTGTACTGCATA	*ramA* qRT PCR-R
KHQ159	GACCAGCGACGGTATCAAATTC	*acrA* qRT PCR-F
KHQ160	GAAGATGGCGCGCAAAGTA	*acrA* qRT PCR-R

KHQ161	GGCGTGAGCGTTGAGAAGTC	*acrB* qRT PCR-F
KHQ162	GGCAACGTAATCCGAAATATCC	*acrB* qRT PCR-R
KHQ165	GGCACCTGCAACGGATGT	*marA* qRT PCR-F
KHQ166	TGCGCGATTTCCGTCATT	*marA* qRT PCR-R
KHQ169	GGAAGTGGTCGCTCAGTACCA	*ompF* qRT PCR-F
KHQ170	GCCGTTCAACTGCTTACCTTTAC	*ompF* qRT PCR-R
KHQ145	GTGGTTTCCGTGGTGTTCCT	*corA* qRT PCR-F
KHQ146	TCCACTTCAGTTCCGGCATAA	*corA* qRT PCR-R
4308	ACCGCGGTAAATGCGACTAT	*mgtA* qRT PCR-F
4309	TGCCGCGACTTTCAGACA	*mgtA* qRT PCR-R
7763	TCAGAAAATGATAAGCAGCATAAAAAA	*mgtB* qRT PCR-F
7764	CCCTGACGATGGCTGTT	*mgtB* qRT PCR-R
6970	CCAGCAGCCGCGGTAAT	*rrsH* qRT PCR-F
6971	TTTACGCCCAGTAATTCCGATT	*rrsH* qRT PCR-R

Supplementary References

1. P. I. Fields, E. A. Groisman, F. Heffron, A Salmonella locus that controls resistance to microbicidal proteins from phagocytic cells. *Science* **243**, 1059-1062 (1989).
2. E. Choi *et al.*, Elongation factor P restricts Salmonella's growth by controlling translation of a Mg(2+) transporter gene during infection. *Sci Rep* **7**, 42098 (2017).
3. M. J. Cromie, Y. Shi, T. Latifi, E. A. Groisman, An RNA sensor for intracellular Mg(2+). *Cell* **125**, 71-84 (2006).
4. K. A. Datsenko, B. L. Wanner, One-step inactivation of chromosomal genes in Escherichia coli K-12 using PCR products. *Proc Natl Acad Sci U S A* **97**, 6640-6645 (2000).
